# Os_2_–Os_4_ Switch Controls DNA Knotting and Anticancer Activity

**DOI:** 10.1002/anie.201602995

**Published:** 2016-05-30

**Authors:** Ying Fu, María J. Romero, Luca Salassa, Xi Cheng, Abraha Habtemariam, Guy J. Clarkson, Ivan Prokes, Alison Rodger, Giovanni Costantini, Peter J. Sadler

**Affiliations:** ^1^ Department of ChemistryUniversity of WarwickCoventryCV4 7ALUK; ^2^ Laboratory of Molecular BiologyCenter for Cancer Research, NCIBuilding 37, Room 5011BethesdaMD20892-4264USA; ^3^ Departamento de Química InorgánicaFacultade de QuímicaUniversidade de Santiago de Compostela15782Santiago de CompostelaSpain; ^4^ CIC biomaGUNEPaseo de Miramón 18220009Donostia-San SebastiánSpain

**Keywords:** cancer, DNA, organometallic, osmium, supramolecular

## Abstract

Dinuclear trihydroxido‐bridged osmium–arene complexes are inert and biologically inactive, but we show here that linking dihydroxido‐bridged Os^II^–arene fragments by a bridging di‐imine to form a metallacycle framework results in strong antiproliferative activity towards cancer cells and distinctive knotting of DNA. The shortened spacer length reduces biological activity and stability in solution towards decomposition to biologically inactive dimers. Significant differences in behavior toward plasmid DNA condensation are correlated with biological activity.

Platinum drugs are used in over 50 % of all chemotherapeutic regimens.^1^ The basis for their activity is believed to be mainly due to DNA binding, in particular to changes in DNA conformation.^2^ Resistance to Pt drugs is a clinical drawback that might be overcome by designing new drugs that induce distinctly different conformational changes in DNA.^3^ Multinuclear metal complexes provide a promising strategy for such an approach.^4^

Herein, we consider the design of multinuclear “piano‐stool” organo‐osmium complexes. Half‐sandwich Os^II^ arene complexes exhibit antitumor activity both in vitro and in vivo.^5^ O,O‐chelated Os^II^ complexes, such as [(η^6^‐arene)Os(acac)Cl], undergo rapid hydrolysis to produce not only the aqua adduct, [(η^6^‐arene)Os(acac)(OH_2_)]^+^, but also the hydroxido‐bridged dimer, [(η^6^‐arene)Os(μ^2^‐OH)_3_Os(η^6^‐arene)]^+^, which is inactive against cancer cells.^6^ Early work by Fujita et al., Chi et al., and Therrien et al. demonstrated that macrocyclic polynuclear Pt and Ru complexes can have anticancer activity comparable to cisplatin, possibly through targeting DNA.^7^ However, little is known about the aqueous stability of polynuclear metallacycles and its effect on biological activity.

In this work, we link inert biologically inactive dinuclear hydroxido‐bridged Os^II^ arene units to form active tetra‐nuclear complexes that can induce DNA knotting. We show that the length of the linker is critical for maintaining stability of the tetranuclear assembly in solution, inducing DNA binding and enhancing antiproliferative activity towards human cancer cells. We compare 4,4′‐azopyridine (pap) as a linker with the shorter pyrazine (prz).

Direct addition of either the pap or prz linkers to the hydroxido intermediate afforded tetranuclear Os^II^ products [Os_4_(η^6^‐*p*‐cym)_4_(μ^2^‐OH)_4_(pap)_2_][PF_6_]_4_ (**1**⋅[PF_6_]_4_) and [Os_4_(η^6^‐*p*‐cym)_4_(μ^2^‐OH)_4_(prz)_2_][PF_6_]_4_ (**2**⋅[PF_6_]_4_). Recrystallization from CH_2_Cl_2_/CH_3_OH solutions gave single crystals of **1**⋅[PF_6_]_4_⋅2 CH_2_Cl_2_⋅CH_3_OH and **2**⋅[PF_6_]_4_⋅6 CH_3_OH⋅2 H_2_O, respectively. The X‐ray crystal structures of **1** and **2** are shown in Figures 1 and S1 (Supporting Information). Crystal data and selected bond lengths and angles are listed in Tables S1, S2.

**Figure 1:**
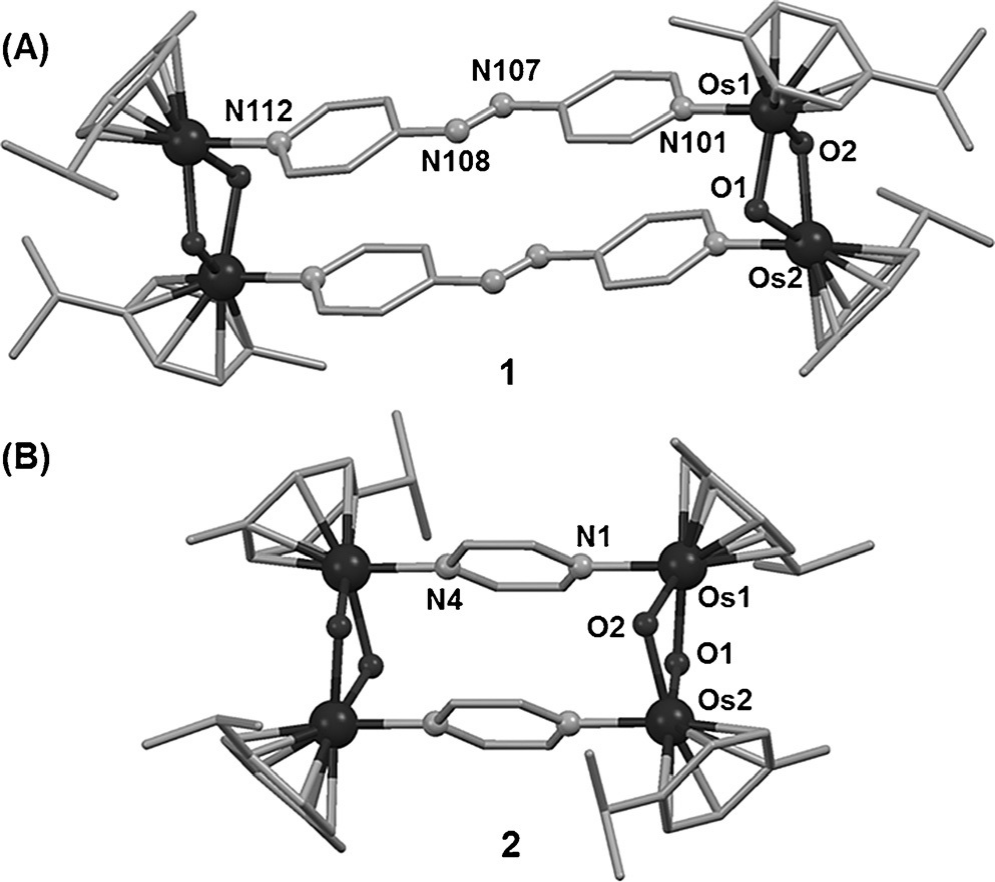
X‐ray crystal structures of **1** (A) and **2** (B).

Very few X‐ray structures of hydroxido‐bridged osmium(II) arene complexes have been reported since the early study of oxo/hydroxido Os^II^ benzene complexes.^8^ The structures of **1**⋅[PF_6_]_4_ and **2**⋅[PF_6_]_4_ (Figure 1) appear to be the first examples of organometallic Os^II^ complexes containing arene, μ^2^‐hydroxido, and aromatic N‐donor ligands simultaneously. A detailed description of the structures is in Figure S1. The distance between the Os atoms bridged by pap is 13.175 Å (**1**), and almost half (6.995 Å) with the prz bridge (**2**). DFT‐optimized geometries are in good agreement with these structures (Tables S3–S5). UV/Vis absorption spectra were recorded for **1** and **2** in acetone (Figure S2). Time‐dependent Density Functional Theory (TDDFT) singlet excited state calculations for both complexes showed that the absorption band at 435–450 nm has ^1^MLCT (metal‐to‐ligand charge‐transfer) character and is composed of two major transitions involving Os‐based occupied orbitals and ligand‐based LUMO and LUMO+1 (Figure S2). Notably, MLCT transitions at wavelengths higher than 600 nm are found for **1**, in agreement with the experimental spectrum. The intensity of such TDDFT transitions is overestimated (particularly the singlet electronic transition S3), as expected for highly delocalized systems displaying charge‐transfer bands.^9^

The stability of the tetramer **1** in solution was investigated first in [D_6_]acetone. The ^1^H NMR spectra of **1**⋅[PF_6_]_4_ at 298 K showed two singlets at 6.68 and 6.66 ppm assignable as OH peaks (Figure S3A). This appears to be the first detection of peaks for bridging‐OH groups in organometallic tetranuclear complexes.^10^ To confirm this assignment, ^1^H NMR at various temperatures and 2D ^1^H DOSY NMR spectra were recorded. The two OH singlets shifted downfield reversibly at lower temperature (Figure S3 and S4) with a linear temperature dependence (Δ*δ*/Δ*T*=−0.009 ppm K^−1^). Interestingly, two peaks assignable to H_2_O and HOD were also observed in these [D_6_]acetone solutions (Figure S5).^11^ When H_2_O (10 μL) was added, the H_2_O and OH bridge peaks shifted downfield (Figure S6). The two low‐field OH peaks disappeared upon addition of D_2_O (Figure S7). The diffusion‐ordered spectroscopy (DOSY) 2D ^1^H NMR spectrum of **1**⋅[PF_6_]_4_ supported assignment of the two singlets (6.68 and 6.66 ppm) to OH bridges (Figure S8). These studies suggested that both OH bridges are involved in H‐bond interactions, which are strongly dependent on temperature and water concentration.

The aqueous stabilities of **1**⋅[PF_6_]_4_ and **2**⋅[PF_6_]_4_ in 10 % MeOD‐d_4_/90 % D_2_O phosphate buffer (1 mm, pH*=7.4) were investigated by ^1^H NMR spectroscopy. After 24 h at 310 K, 63 % of cationic **1** was still present as the intact tetramer, whereas **2** decomposed completely under the same conditions and the solution became colorless (Figure S9). 2D ^1^H DOSY experiments on **2**⋅[PF_6_]_4_ (Figure 2) confirmed the release of the aromatic linker with a concomitant breakdown of the tetranuclear assembly and generation of an hydroxido‐bridged Os^II^–arene dimer [Os_2_(η^6^‐*p*‐cym)_2_(μ^2^‐OH)_3_]^+^ for both **1** and **2**. The formation of this biologically inert hydroxido dimer^6^ was confirmed by ESI‐MS (Figure S9).

**Figure 2:**
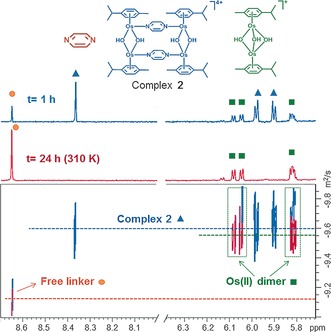
2D DOSY ^1^H NMR study of the stability of complex **2**⋅[PF_6_]_4_ in buffered aqueous solution (see the Supporting Information); t=time point at which spectra were recorded.

The antiproliferative activity of **1** and **2** towards cancer cells was determined. The IC_50_ value for A2780 human ovarian cancer cells decreased dramatically from >100 μm to 10 μm as the linker length increased from pyrazine in complex **2**⋅[PF_6_]_4_ to 4,4′‐azopyridine in complex **1**⋅[PF_6_]_4_ (Table 1). An analogous trend in antiproliferative activity was observed for A549 and H596 human non‐small‐cell lung cancer (NSCLC) cell lines (Table 1). In these two cell lines, **1**⋅[PF_6_]_4_ showed similar anticancer potency to cisplatin and was approximately 2‐times more potent than **2**⋅[PF_6_]_4_. No antiproliferative activity was observed for the free linker ligands. The highest antiproliferative activity is therefore associated with the cationic complex that more readily retains its tetranuclear structure in solution (complex **1**). The anticancer activity of **2**⋅[PF_6_]_4_ is comparable to some reported dinuclear Ru^II^ cylinders.^12^

**Table 1:** Cytotoxicity (IC_50_, μm) of **1**⋅[PF_6_]_4_ and **2**⋅[PF_6_]_4_ towards A549 (lung), H596 (lung), and A2780 (ovarian) human cancer cell lines.

Complex	A549	H596	A2780
CDDP	6.7(±0.6)	5.9(±1.4)	1.8(±0.1)
**1**	5.2(±0.3)	4.8(±2.0)	10.1(±0.1)
**2**	12.9(±2.2)	10.8(±1.3)	>100

Metallosupramolecules are also known to bind in the grooves of DNA.^13^ Therefore, binding of complexes **1** and **2** to calf thymus DNA (ct‐DNA) was also investigated (Figures S11 and S13). The characteristic circular dichroism (CD) bands of ct‐DNA (Figure S11) and the negative linear dichroism (LD) band arising from the DNA bases (Figure S13) decreased in intensity with increasing concentrations of **1** or **2**, even at low Os loading. Red fibers, likely corresponding to DNA‐tetramer adducts, were observed at high Os loading (Figure S12). These data suggest that both Os^II^ tetramers bind to ct‐DNA and induce DNA condensation, facilitated by their 4+ charge, but more significantly for tetramer **1**.

Further insight into Os^II^ tetramer–DNA interactions was obtained using tapping mode atomic force microscopy (AFM) (Figures 3 and S14). AFM images were recorded at two different time points, 30 min after preparing the samples and 24 h after incubation at 310 K. Figure 3 a, b shows images of the control sample where the free pBR322 plasmid was deposited onto freshly cleaved mica treated with Mg^2+^ to ensure firm adsorption of the negatively charged DNA. Mainly relaxed open circular (OC) and linear (L) forms were observed on the surface after the free plasmid relaxation procedure (Supporting Information). Analysis of cross sections of the different forms resulted in comparable heights of 0.70±0.08 nm (OC) and 0.70±0.10 nm (L; Figure 3 a,b). These values are smaller than the theoretical DNA diameter (ca. 2 nm), probably owing to electrostatic interactions with the positively charged mica surface that tend to compress the plasmids.^14^

**Figure 3:**
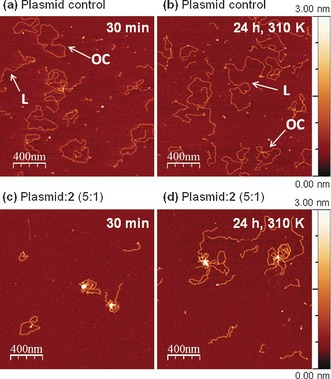
AFM images of pBR322 plasmid DNA (a, b) in the absence, and (c, d) the presence of **2**⋅[PF_6_]_4_ in a DNA base:tetramer ratio of 5:1. Images were recorded 30 min after preparing the samples (a, c) and 24 h after incubation at 310 K (b, d). OC=open‐circular; L=linear plasmid DNA. Conditions: 2 mm HEPES buffer, pH 7.2, 0.9 mm MgCl_2_, [plasmid]=1.6 μg mL^−1^.

Figures 3 c, d and S14 show the effect of interactions between plasmid DNA and complexes **1** or **2** at a DNA base:Os_4_ ratio of 5:1. No plasmids were detected on any of the analyzed samples after mixing with the more biologically active tetramer **1**⋅[PF_6_]_4_ (Figure S14a, b). This was interpreted as an indication that the DNA forms an adduct with cationic tetramer **1**, thus becoming less negatively charged and less strongly bound to the positively charged mica substrate.

Interaction with the less biologically active tetramer **2**⋅[PF_6_]_4_ gave rise to the formation of knotted aggregates of plasmid DNA, appearing as plasmid loops emerging from a condensed nucleus, probably generated by the linking of several DNA strands mediated by **2** (Figure 3 c). The DNA adducts with complex **2** were strongly attached to the Mg^2+^‐treated surface. This suggests a larger negative charge density of DNA‐**2** with respect to DNA‐**1** adducts, probably owing to a reduced number of bound molecules of **2** with respect to **1**. A cross‐sectional height analysis confirmed this conclusion (Figure S14, detailed discussion in the Supporting Information). This is the first report on the formation of this type of DNA aggregate with organo‐osmium anticancer complexes. The unusual behavior of **1** and **2** differs considerably from the DNA cross‐linking caused by Ru^II^–arene μ‐Hoxonato tetramers^15^ or the DNA full‐coiling induced by metallocylinders.^12^

Experiments carried out at a lower DNA:**2** mol ratio (2.5:1) showed the formation of similar knotted aggregates (Figure S14f), characterized by a higher surface density and a slightly larger cross‐sectional height (Supporting Information), confirming a higher linear density of **2** bound to the DNA strands. Conversely, a lower density of bridging tetramers **2** was observed on incubating 5:1 DNA:**2** samples for 24 h at 310 K (Figures 3 d and S14e, detailed discussion in the Supporting Information). ^1^H NMR experiments demonstrated that incubation caused cleavage of the tetranuclear structure of these Os^II^ complexes into dimers (Figures 2 and S9), and can thus be expected to partially release plasmids from the central core in the DNA‐**2** adducts, resulting in the larger loops observed in Figure 3 d. Both AFM experiments were consistent with the dichroism measurements, indicating that the changes in the CD and LD signals observed upon increasing concentrations of **2** (Figures S11 and S13, respectively) or incubating the 5:1 DNA:**2** samples (Figure S13) are probably due to a different extent of DNA condensation.

In conclusion, our studies reveal how the choice of spacer length in tetranuclear organo‐osmium metallacycles can control their stability in solution and their biological activity, which correlates with their ability to induce plasmid DNA condensation. The more cytotoxic tetramer **1** reduces the DNA negative charge significantly more than tetramer **2**, although the latter also exerts a considerable effect on DNA structure. The different modes of interaction of **1** and **2** with DNA seem likely to contribute to the large difference in cytotoxicity exhibited by these candidate metallodrugs and may play a role in their anticancer mechanism of action.

## Supporting information

As a service to our authors and readers, this journal provides supporting information supplied by the authors. Such materials are peer reviewed and may be re‐organized for online delivery, but are not copy‐edited or typeset. Technical support issues arising from supporting information (other than missing files) should be addressed to the authors.

Supplementary
