# Supplementary material for: Os2–Os4 Switch Controls DNA Knotting and Anticancer Activity
Source: Angew Chem Int Ed Engl. 2016 May 30;55(31):8909–12. doi: 10.1002/anie.201602995 (PMC4982093; doi:10.1002/anie.201602995)
Supplement: Supplementary file 1 — Supplementary [file ANIE-55-8909-s001.pdf]

Angewandte Chemie
Eine Zeitschrift der Gesellschaft Deutscher Chemiker

Supporting Information

Os_2–Os_4 Switch Controls DNA Knotting and Anticancer Activity

*Ying Fu[+], María J. Romero[+], Luca Salassa, Xi Cheng, Abraha Habtemariam, Guy J. Clarkson, Ivan Prokes, Alison Rodger, Giovanni Costantini, and Peter J. Sadler**

anie_201602995_sm_miscellaneous_information.pdf

Supporting Information

Abbreviations:

4-4`-azopyridine: pap; pyrazine: prz.

Contents:

Experimental section

Scheme S1

Tables S1-S9

Figures S1-S15

Discussion of AFM results:

1. *AFM analysis of the pBR322 plasmid DNA*
2. *AFM analysis of the interaction of plasmid DNA with tetramer* ***1***
3. *AFM analysis of the interaction of plasmid DNA with tetramer* ***2***
4. *AFM analysis of the interaction of DNA with pre-incubated tetramers* ***1*** *and* ***2***

Experimental

Materials. $OsCl_3 \cdot 3H_2O$ was purchased from Alfa-Aesar. Ethanol and methanol were dried over Mg/I_2 or anhydrous quality was used (Aldrich). All other reagents used were obtained from commercial suppliers and used as received. The preparation of the starting materials $[Os(\eta^6\text{-}p\text{-cym})Cl_2]_2$ (where *p*-cym is *para*-cymene) have been previously reported.[1] 4,4'-Azopyridine (pap) and pyrazine (prz), calf-thymus DNA (ct-DNA) and pBR322 plasmid DNA were purchased from Sigma-Aldrich. The A2780 human ovarian carcinoma cell line was purchased from European Collection of Animal Cell Cultures (Salisbury, UK), A549 (adenocarcinomic human alveolar basal epithelial cells) and H596 (human adenosquamous carcinoma cells) from ATCC (American Type Culture Collection, USA). RPMI-1640 media and trypsin were purchased from Invitrogen, bovine serum from Biosera, penicillin, streptomycin, trichloroacetic acid (TCA) and sulforhodamine B (SRB) from Sigma-Aldrich, and tris[hydroxymethyl]aminomethane from Formedium.

Instrumentation and methods

NMR Spectroscopy. ^1H NMR spectra were acquired in 5 mm NMR tubes at 298 K on either Bruker DPX-400, Bruker DRX-500 or AVIII-600 spectrometers using acetone-d_6 or 10% MeOD-d_4/90% D_2O phosphate buffer (1 mM, pH* 7.4) solutions as indicated. ^1H NMR chemical shifts were referenced to acetone-d_6 (2.09 ppm). All data processing was carried out using TOPSPIN version 2.5 (Bruker U.K. Ltd.). ^1H Diffusion-ordered Spectroscopy (DOSY) experiments for **1**·$[PF_6]_4$ (1 mM in acetone-d_6 or 0.4 mM in 10% MeOD-d_4/90% D_2O sodium cacodylate buffer (1 mM, pH* 7.4) and **2**·$[PF_6]_4$ (0.4 mM in 10% MeOD -d_4/90% D_2O cacodylate buffer (1 mM, pH* 7.4)) were performed on the AV500 spectrometer at 298 K.

In order to study the stability of complexes **1**·$[PF_6]_4$ and **2**·$[PF_6]_4$ in aqueous solution, 2D DOSY ^1H NMR was employed. Spectra were recorded at t = 1 h after dissolution of the complex and t = 24 h after incubation at 310 K in 10% MeOD-d_4/90% D_2O sodium cacodylate buffer (1 mM, pH* 7.4). Assignments in Figure 2 (main script): complex **2**·$[PF_6]_4$ (purple triangle), free linker (orange circle) and Os^{II} arene dimer (green square); these species have markedly different diffusion coefficients (m^2/s, y axis). In a similar experiment with complex **1**·$[PF_6]_4$, no new diffusion signals were observed after 1 h (see Fig. S10), indicative of its higher stability.

Elemental Analysis. Elemental analysis (carbon, hydrogen and nitrogen) was carried out through Warwick Analytical Service using an Exeter analytical elemental analyzer (CE440).

Electrospray Ionization Mass Spectrometry (ESI-MS). Spectra were obtained by preparing the samples in methanol or 10% methanol/90% water and infusing into the mass spectrometer (Bruker Esquire 2000). The mass spectra were recorded with a scan range of m/z 500-1000 or 800-2000 for positive ions. Data were processed using Data Analysis version 3.3 (Bruker Daltonics). The formation of the hydroxido dimeric complex from complexes **1**·$[PF_6]_4$ and **2**·$[PF_6]_4$ was confirmed by ESI-MS with the detection of appropriate peaks ($[Os_2(p\text{-cym})_2(OH)_3]^+$, 701.1 m/z; $[Os_2(p\text{-cym})_2(OH)_4+Na]^+$, 743.0 m/z) from aqueous solutions.

UV-Visible Absorption Spectroscopy. UV-visible absorption spectra were recorded on a Cary 300-Bio spectrophotometer using 1-cm path length quartz cuvettes (0.5 mL) and a PTP1 Peltier temperature controller. Spectra of **1**$[PF_6]_4$ and **2**$[PF_6]_4$ were recorded at *ca*. 310 K in methanol or acetone. The DNA concentration (mol of bases/L) was determined by absorption spectroscopy using the molar extinction coefficient at the maximum absorption wavelength (λ_{max}= 260 nm, ε = 6600 $cm^{-1}\cdot mol^{-1}\cdot dm^3$). The purity of the DNA stock solutions (in sodium cacodylate buffer or HEPES buffer at pH 7.2) was assessed by calculating the ratio $A_{260/280}$ > 1.8, which confirmed that the DNA was free of protein.

pH* Measurements. pH* (pH meter reading from D_2O solution without correction for effects of deuterium on glass electrode) values were measured at ambient temperature before the NMR spectra were recorded, using a Corning 240 pH meter equipped with a microcombination electrode calibrated with Aldrich buffer solutions at pH 4, 7 and 10.

X-ray Crystallography. X-ray diffraction data of **1**·$[PF_6]_4\cdot 2CH_2Cl_2\cdot CH_3OH$ and **2**·$[PF_6]_4\cdot 6CH_3OH\cdot 2H_2O$ were obtained on an Oxford Diffraction Gemini four-circle system with a Ruby CCD area detector using Mo Kα radiation.[2] Absorption corrections were applied using ABSPACK. The crystals were mounted in oil and held at 100(2) K with the Oxford Cryosystem Cryostream Cobra. The structures were solved by direct methods using Olex2 and SHELXS (TREF) with additional light atoms found by Fourier methods.[3] Refinement used SHELXL 97.[4] Hydrogen atoms were placed at geometrically calculated positions and refined riding on their parent atoms. Anisotropic displacement parameters were used for all non-H atoms; H-atoms were given isotropic displacement parameter equal to 1.2 (or 1.5 for methyl H-atoms) times the equivalent isotropic displacement parameter of the atom to which they are attached.

In case of complex **1**·$[PF_6]_4\cdot 2CH_2Cl_2\cdot CH_3OH$, no hydrogens were located on the methanol but were included in the formula so as to calculate the correct density. The structure contains a molecule of dichloromethane modelled as disordered over two positions with the occupancy allowed to refine then fixed at 50:50. The *p*-cymene ligand C201-C210 was modelled as disordered over two positions (C202-C210 and C21A-C30A) by a small rotation around the bond to Os1 and refine to a ratio of 47:53. *p*-Cymene ligand C301-C310 had disorder in the isopropyl group modelled as disordered over two positions in the ratio of 56:44. One PF_6^- counterion (P10-F16) was modelled as disordered over two positions and refined to a ratio of 75:25. The minor component P10A-F16A was refined isotropically. The other PF_6^- counterion was also disordered over two positions but this was complicated by a molecule of methanol that had the carbon siting on an inversion centre. As this methanol was very close to one of the disordered PF_6^- molecules, a model was developed where both a half occupied PF_6^- P20-F26 and the symmetry related disordered component P20A-F26A at half occupancy and a partially occupied methanol (25% occupancy) where contained in a Part-1. This gave a refinement that converged and gave chemically sensible distances between the disordered components. Drawings of crystal structures were made with Ortep-3 and Mercury 2.4.

For complex **2**·$[PF_6]_4\cdot 6CH_3OH\cdot 2H_2O$, the hydrogens were located on the bridging hydroxides O1 and O2 in a difference map and their positions were allowed to refine freely but given thermal parameters equal to 1.5 times the atom they were attached. The hydrogens on the water O33 were located in a difference map and refined with DFIX and DANG restraints to give the appropriate O-H bond lengths and given thermal parameters U_{iso} 1.5

times the U_{equiv} of the parent oxygen. The unlocated hydrogens were placed in the formula so as to give the correct calculated density. The P20 atom was disordered with two orientations about the phosphorus atom and the occupancy of the two components was linked to a free variable which refined to a ratio 77:23 major:minor. The minor component was refined isotropically.

Crystallographic data have been deposited in CIF format in the Cambridge Crystallographic Data Centre (https://www.ccdc.cam.ac.uk/): CCDC 1059768 = complex **1**, CCDC 1059767 = complex **2**.

Cell Cultures. A2780, A549 and H596 cells were cultured in RPMI 1640 cell culture medium supplemented with 1 mM sodium pyruvate, 2 mM L-glutamine and 10% fetal bovine serum (all from Sigma).

Determination of IC_{50} Values. The concentrations of the osmium complexes that inhibit 50% of the proliferation of cancer cells were determined using the sulforhodamine B assay.[5] A2780 cells were seeded in 96-well plate (Falcon) at 5000 cells/well, and incubated for 48 h before the treatment. The complexes were solubilised in DMSO to provide 10 mM stock solutions. These were serially diluted by cell culture media to give concentrations four-fold greater than the final concentrations for the assay. The complexes diluted in cell culture media were added to the 96-well plate with cells in triplicate. The final DMSO concentration in each well was no more than 1% (v/v). The media containing the complexes were removed after 24 h. The cells were washed with phosphate buffered saline once and cell culture medium was added (150 µL/well). The cells were then allowed to grow for a further 72 h. The surviving cells were fixed by adding 150 µL/well of 50% (w/v) trichloroacetic acid and incubated for 1 h in the fridge (277 K). The plates were washed with tap water three times and dried under a flow of warm air, 0.4% sulforhodamine B solution (100 µL/well) was added, followed by washing with 1% acetic acid five times and drying under a flow of warm air. The dye was dissolved in 10 mM Tris buffer (200 µL/well). The absorbance of each well was determined using a Thermo Multiskan Ascent plate reader (Labsystems) at 540 nm. The absorbance of SRB in each well is directly proportional to the cell number. Then the absorbance was plotted against concentration and the IC_{50} determined by using Origin software.

Circular and Linear Dichroism (CD and LD). CD/LD spectra of ct-DNA (200 µM in bases) modified by the two tetranuclear osmium complexes were recorded at ambient temperature (ca. 293 K) on a CD Jasco J-815 spectropolarimeter adapted for LD spectroscopy. Complexes **1**$[PF_6]_4$ and **2**$[PF_6]_4$ are achiral and do not exhibit intrinsic CD signals.

DNA-metal complex adducts were prepared as follows: an appropriate volume of the osmium complex (stock solution was 400 µM in 5% DMSO/95% sodium cacodylate buffer (1 mM), pH 7.2) was added to a 200 µM ct-DNA solution (dissolved in 1 mM sodium cacodylate buffer, 20 mM NaCl, pH 7.2) at different ratios, keeping the ct-DNA concentration constant in all the experiments. The ct-DNA base to metal complex mol ratios were 20:1, 10:1, 5:1 and 2.5:1). CD spectra were recorded at 293 K at 200 nm/min, data integration time 1 s and 4 accumulated scans, in a quartz cuvette with 0.5 cm pathlength.

Linear dichroism (LD) is directly related to the orientation of DNA relative to the helix axis. This technique was used to study the geometry of DNA adducts of cationic osmium tetramers

1 and **2**.[6] Changes in the intensity of the negative LD band at 260 nm might result from either an increase in DNA flexibility or a shortening of the DNA by kinking, bending, compaction or aggregation.[7] The LD experiments were performed with a small volume quartz LD cell with a rotating outer quartz cylinder of internal diameter of 2.9 mm, and stationary inner cylinder, a 2.4 mm diameter quartz rod.[8] The rotation speed used in the experiments was 3000 rpm.

Atomic force microscopy (AFM). Topographic images of pBR322 plasmid DNA (4363 bp) with tetramers **1**·$[PF_6]_4$ or **2**·$[PF_6]_4$ were collected on a Veeco Multimode V instrument with a Nanoscope V controller operating in tapping-mode. All solutions were prepared with 18.2 MΩ·cm Milli-Q water filtered through 0.2 nm filters and centrifuged once (8000×g) to avoid salt deposits in order to provide a clear background for AFM images. Plasmid DNA stock solutions were kept at 253 K. $MgCl_2$ was employed at a concentration of 0.9 mM in H_2O to increase attachment of negatively-charged plasmid molecules to the mica surface.[9] Open-circular (OC) and linear (L) plasmid molecules were obtained after incubating a solution of supercoiled pBR322 plasmid DNA (2.5 μg/mL) in HEPES buffer (2 mM, pH 7.2) at 333 K for 30 min.[10] DNA-osmium complex adducts were prepared by adding an appropriate volume of the Os(II) complex in HEPES buffer (DNA base to metal complex ratio 5:1 or 2.5:1 as indicated) to the incubated plasmid DNA (final plasmid concentration = 1.6 μg/mL) in 2 mM HEPES buffer (pH 7.2). Samples were prepared for AFM by placing a drop (12 μl) of plasmid DNA solution (1.6 μg plasmid/mL) or DNA-metal complex solution onto freshly cleaved mica (9.9 mm diameter, Agar Scientific Ltd.) pre-treated with $MgCl_2$ (0.9 mM aqueous solution) for a firm adsorption of the negatively charged DNA. After adsorption for 5 min at room temperature, the samples were gently rinsed for 10 s with 18.2 MΩ·cm Milli-Q water directed onto the mica surface using a squeeze bottle. The samples were blow-dried with compressed dry N_2 and the images were obtained in air at room temperature (ca. 293 K). The cantilevers used for imaging were Bruker FMV Si probes (k ~ 2.8 N/m, nominal tip radius 10-12 nm). Two samples from each mixture were imaged in several places to obtain reliable measurements at two different DNA base-to-metal complex ratios. The control plasmid was also imaged for each experiment under the same experimental conditions. Images were processed using WSxM software.[11]

Computation. All calculations were performed with the Gaussian 03 (G03) program[12] employing the DFT method, Becke three parameter hybrid functional and Lee-Yang-Parr's gradient corrected correlation functional (B3LYP).[13] The LanL2DZ basis set[14] and effective core potential were used for the Os atom and the 6-31G basis set[15] was used for all other atoms. Geometry optimizations of complexes **1**·$[PF_6]_4$ and **2**·$[PF_6]_4$ in the ground state were performed in the gas phase and the nature of all stationary points was confirmed by normal mode analysis. The conductor-like polarizable continuum model method (CPCM)[16] with acetone as solvent was used to calculate the electronic structure and the excited states of **1**·$[PF_6]_4$ and **2**·$[PF_6]_4$ in solution. One-hundred and twenty singlet excited states and the corresponding oscillator strengths were determined with a Time-dependent Density Functional Theory (TDDFT)[17] calculation. The electronic distribution and the localization of the singlet excited states were visualized using the electron density difference maps (EDDMs).[18] GaussSum 1.05[19] was used for EDDMs calculations.

Synthesis and characterization

(**1**) $[Os_4(\eta^6\text{-}p\text{-cym})_4(OH\cap OH)_2(pap)_2][PF_6]_4$

$AgNO_3$ (85.6 mg, 0.51 mmol) was added to a suspension of $[Os(\eta^6\text{-}p\text{-cym})Cl_2]_2$ (100 mg, 0.13 mmol) in 10 mL of water. The suspension was stirred overnight at ambient temperature. A white precipitate was filtered off giving a yellow clear solution. When 4,4'-azopyridine (23.2 mg, 0.13 mmol) was added, the color changed from yellow to red immediately. The red solution was stirred overnight at ambient temperature. NH_4PF_6 (205.4 mg, 1.26 mmol) was added to the red solution and a brown-red precipitate was collected, washed with methanol and diethyl ether and finally dried *in vacuo*. Yield 94.4 mg (63 %). ^1H NMR (MeOD-d_4) δ 8.39 (d, 8H, J = 7 Hz), 7.56 (d, 8H, J = 7 Hz), 5.82 (dd, 16H), 2.32-2.25 (m, 4H), 2.37 (s, 12H), 1.06 (d, 24H, J = 6 Hz) ppm. CHN analysis, Found: C, 31.27%; H, 3.25%; N, 4.68%, Calcd for $C_{60}H_{76}F_{24}N_8O_4Os_4P_4$: C, 31.14%; H, 3.31%; N, 4.84%. ESI^+-MS (MeOH, *m/z*): 1546.1 $[Os_4(p\text{-cym})_4(OH)_4(pap)\text{-}3H]^+$. Single crystals of **1**$\cdot[PF_6]_4\cdot 2CH_2Cl_2\cdot CH_3OH$ suitable for the X-ray analysis were obtained from dichloromethane-methanol solutions.

(**2**) $[Os_4(\eta^6\text{-}p\text{-cym})_4(OH\cap OH)_2(prz)_2][PF_6]_4$

$AgNO_3$ (85.6 mg, 0.51 mmol) was added to a suspension of $[Os(\eta^6\text{-}p\text{-cym})Cl_2]_2$ (100 mg, 0.13 mmol) in 10 mL of water. The suspension was stirred overnight at ambient temperature. A white precipitate was filtered off resulting in a clear yellow solution. When pyrazine (10.0 mg, 0.13 mmol) was added, the color changed from yellow to red immediately. The red solution was stirred overnight at ambient temperature. NH_4PF_6 (205.4 mg, 1.26 mmol) was added and the red precipitate was collected and washed with methanol and diethyl ether, and finally dried *in vacuo*. Yield 85.3 mg (62%). ^1H NMR (MeOD-d_4) δ 8.34 (s, 8H), 6.00 (dd, 16H, J = 6 Hz), 4.87 (s, 12H), 2.59-2.53 (m, 4H), 1.24 (d, 24H, J = 6 Hz) ppm. CHN analysis, Found: C, 26.79%; H, 3.12%; N, 2.69%, Calcd for $C_{48}H_{68}F_{24}N_4O_4Os_4P_4$: C, 27.38%; H, 3.25%; N, 2.66%. ESI^+-MS (MeOH, *m/z*): 1546.1 $[Os_4(p\text{-cym})_4(OH)_4(prz)_2\text{-}4H\text{+}Na]^+$, 860.5 $[Os_2(p\text{-cym})_2(OH)_3(prz)_2]^+$. Single crystals of **2**$\cdot[PF_6]_4\cdot 6CH_3OH\cdot 2H_2O$ suitable for the X-ray analysis were obtained from dichloromethane-methanol solutions.

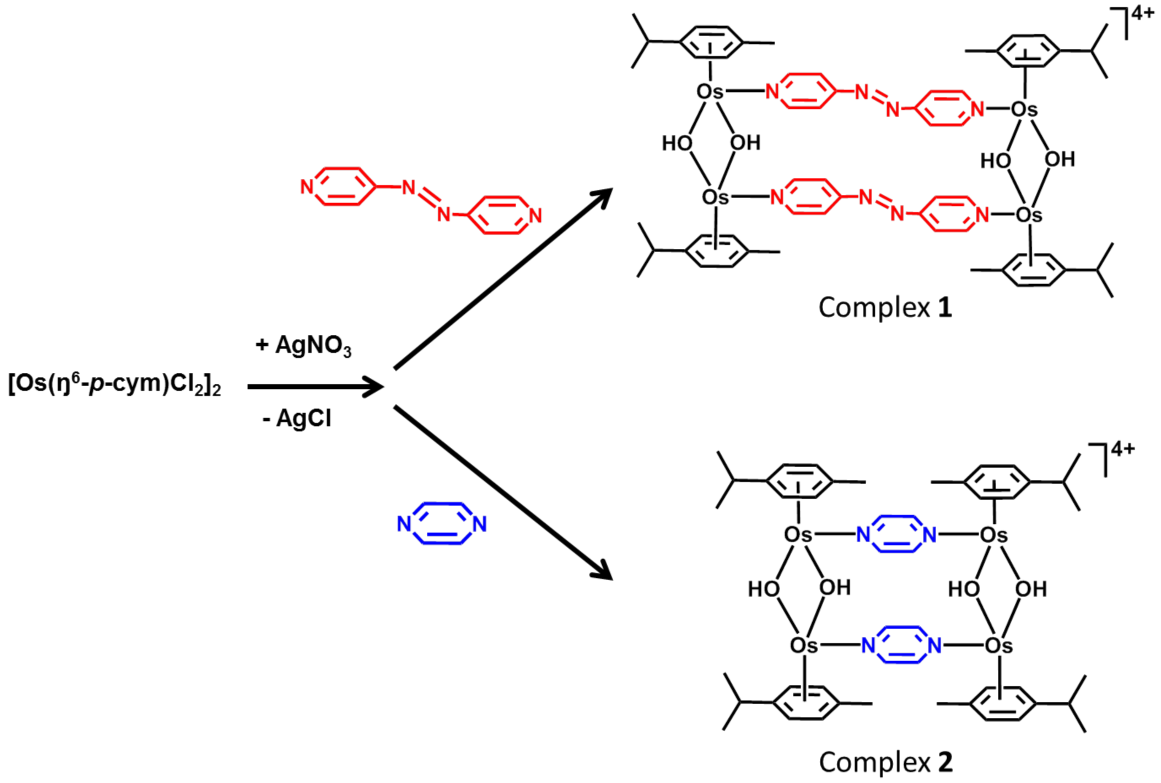

Scheme S1. Synthesis of [Os_4(η^6-*p*-cym)$_4$(OH∩OH)$_2$(pap)$_2$][PF_6]$_4$ (**1**·[PF_6]$_4$) and [Os_4(η^6-*p*-cym)$_4$(OH∩OH)$_2$(prz)$_2$][PF_6]$_4$ (**2**·[PF_6]$_4$).

Table S1. X-ray crystallographic data and structure refinement parameters for complexes **1**·$[PF_6]_4 \cdot 2CH_2Cl_2 \cdot CH_3OH$ and **2**·$[PF_6]_4 \cdot 6CH_3OH \cdot 2H_2O$.

	**1**·$[PF_6]_4 \cdot 2CH_2Cl_2 \cdot CH_3OH$	**2**·$[PF_6]_4 \cdot 6CH_3OH \cdot 2H_2O$
Empirical Formula	$C_{63}H_{80}Cl_4F_{24}N_8O_5Os_4P_4$	$C_{54}H_{96}F_{24}N_4O_{12}Os_4P_4$
Formula weight	2511.83	2334.02
Crystal system	Monoclinic	Monoclinic
Crystal size / mm	0.12 x 0.10 x 0.05	0.30 x 0.30 x 0.10
Space group	P2(1)/c	P2(1)/n
Crystal	orange block	red block
a / Å	12.7866(2)	14.09796(15)
b / Å	14.3624(2)	16.15655(16)
c / Å	22.1980(4)	16.96055(17)
α / deg	90.00	90.00
β / deg	98.152(2)	91.0734(9)
γ / deg	90.00	90.00
Volume / $Å^3$	4035.39(11)	3862.50(7)
Temperature / K	100(2)	100(2)
Z	2	2
μ [mm^{-1}]	6.596	6.753
Reflections collected	22514	80658
Independent reflections [R_{int}]	10176 [0.0328]	10022 [0.0456]
Data/ restraints/ parameters	10176/ 192/ 725	10022/ 75/ 509
R_1[a], wR_2[b] [$I > 2\sigma(I)$]	0.0367, 0.0759	0.0275, 0.0691
R_1[a], wR_2[b] (all data)	0.0490, 0.0826	0.0374, 0.0709
GOF[c]	1.035	1.072
Δρ max and min/ $eÅ^{-3}$	1.926 and -1.566	2.73 and -1.03

[a] $R_1 = \Sigma||Fo| - |Fc||/\Sigma|Fo|$.

[b] $wR_2 = [\Sigma w(Fo^2 - Fc^2)^2/\Sigma wFo^2)]^{1/2}$.

[c] $GOF = [\Sigma w(Fo^2 - Fc^2)^2/(n-p)]^{1/2}$

where n = number of reflections and p = number of parameters

Table S2. Selected bond lengths (Å) and angles (deg) for **1**·$[PF_6]_4$·$2CH_2Cl_2$·CH_3OH (red) and **2**·$[PF_6]_4$·$6CH_3OH$·$2H_2O$ (blue). The atom numbering is indicated below. Symmetry related atoms are labelled with superscripts "ii" and "i" respectively.

**1**·$[PF_6]_4$·$2CH_2Cl_2$·CH_3OH		**2**·$[PF_6]_4$·$6CH_3OH$·$2H_2O$	
Os(1)−O(1)	2.114(4)	Os(1)−O(1)	2.101(3)
Os(1)−O(2)	2.093(3)	Os(1)−O(2)	2.087(2)
Os(2)−O(1)	2.107(3)	Os(2)−O(1)	2.106(2)
Os(2)−O(2)	2.111(3)	Os(2)−O(2)	2.096(3)
Os(1)−Arene centroid	1.653	Os(1)−Arene centroid	1.660
Os(1)−Os(2)	3.334	Os(1)−Os(2)	3.309
Os(1)−Os(2^{ii})	13.175	Os(1)−Os(2^{i})	6.995
Os(1)−N(101)	2.099(4)	Os(1)−N(1)	2.110(3)
Os(2^{ii})−N(112)	2.110(4)	Os(2^{i})−N(4)	2.107(3)
O(2)−Os(1)−O(1)	73.31(13)	O(2)−Os(1)−O(1)	73.35(10)
Os(1)−O(1)−Os(2)	104.37(15)	Os(1)−O(1)−Os(2)	103.72(11)
Os(1)−O(2)−Os(2)	104.95(15)	Os(1)−O(2)−Os(2)	104.58(11)
O(2)−Os(2)−O(1)	73.09(13)	O(2)−Os(2)−O(1)	73.08(10)

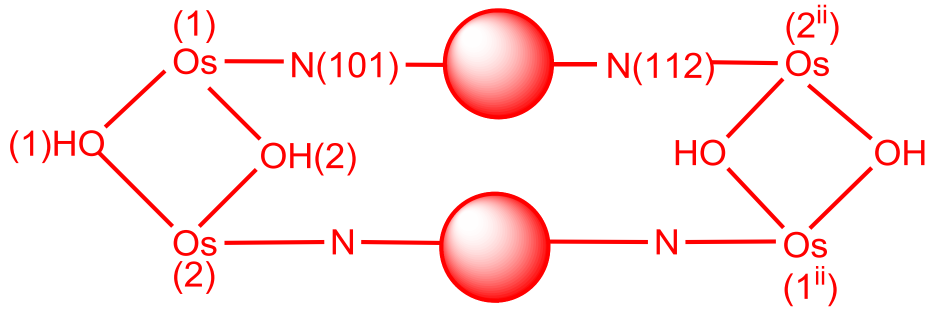

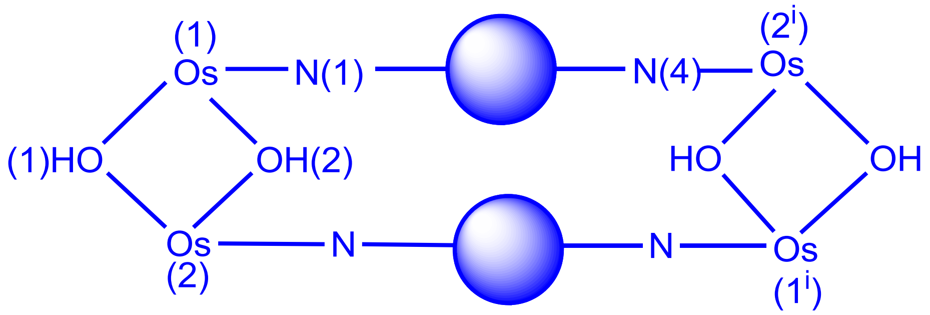

Table S3. Selected DFT (B3LYP/LanL2DZ/6-31G) bond lengths (Å) and angles (deg) for complexes **1**·$[PF_6]_4$ (red) and **2**·$[PF_6]_4$ (blue). The atom numbering is indicated below. Symmetry related atoms are labelled with superscripts "ii" and "i" respectively. DFT-optimized geometries are in good agreement with the experimental structures. Bond distances are only slightly overestimated (ca. 0.03 Å).

**1**·$[PF_6]_4$		**2**·$[PF_6]_4$	
Os(1)−O(1)	2.142	Os(1)−O(1)	2.143
Os(1)−O(2)	2.134	Os(1)−O(2)	2.147
Os(2)−O(1)	2.138	Os(2)−O(1)	2.146
Os(2)−O(2)	2.142	Os(2)−O(2)	2.143
Os(1)−Os(2)	3.458	Os(1)−Os(2)	3.446
Os(1)−Os(2^{ii})	13.275	Os(1)−Os(2^{i})	6.981
Os(1)−N(101)	2.099	Os(1)−N(1)	2.082
Os(2^{ii})−N(112)	2.087	Os(2^{i})−N(4)	2.083
O(2)−Os(1)−O(1)	70.859	O(2)−Os(1)−O(1)	70.988
Os(1)−O(1)−Os(2)	107.765	Os(1)−O(1)−Os(2)	106.922
Os(1)−O(2)−Os(2)	107.930	Os(1)−O(2)−Os(2)	106.857
O(2)−Os(2)−O(1)	70.782	O(2)−Os(2)−O(1)	70.996

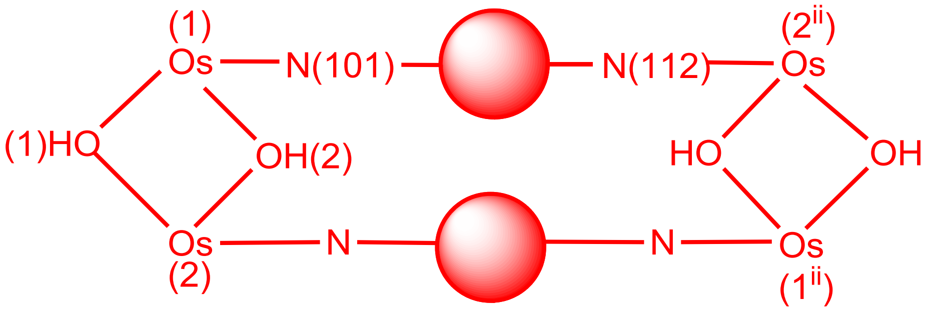

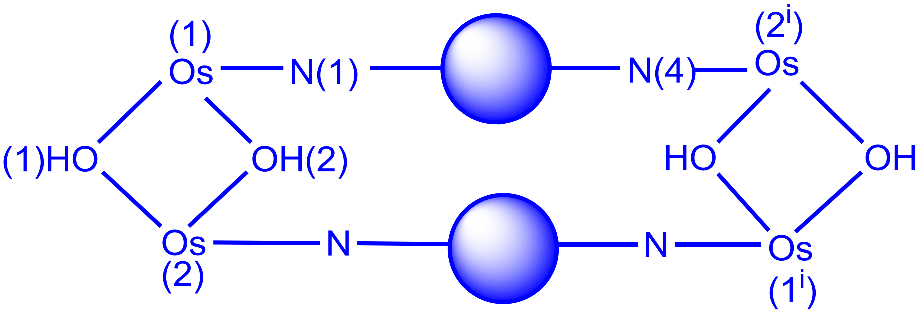

Table S4. Selected frontier orbitals of **1**·$[PF_6]_4$ calculated in acetone (B3LYP/LanL2DZ/6-31G, CPCM). Tetramer **1** has an HOMO orbital of Os-arene character and a LUMO centered on the linker ligand.

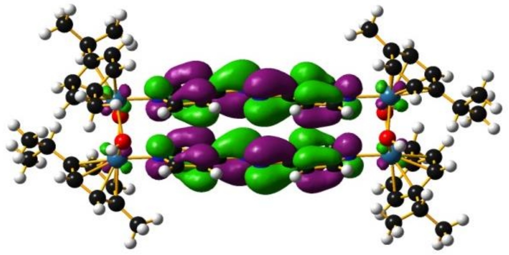 LUMO+1	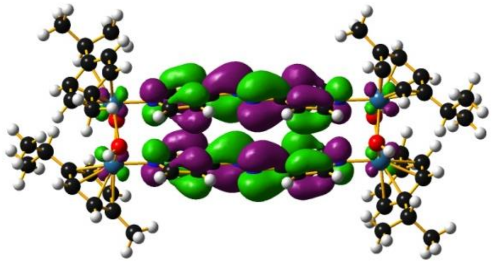 LUMO
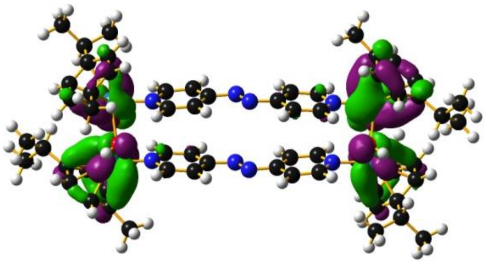 HOMO	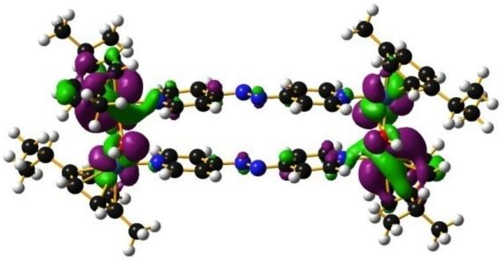 HOMO−2

Table S5. Selected frontier orbitals of **2**·$[PF_6]_4$ calculated in acetone (B3LYP/LanL2DZ/6-31G, CPCM). Tetramer **2** has an HOMO orbital of Os-arene character and a LUMO centered on the linker ligand.

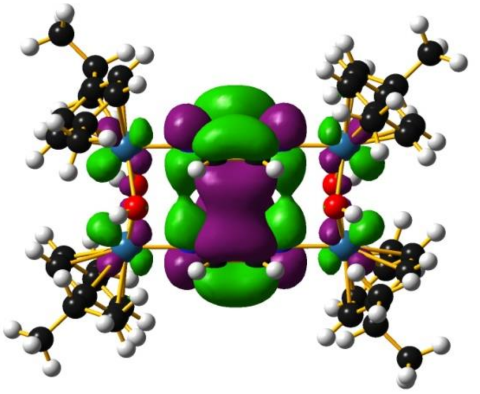 LUMO	
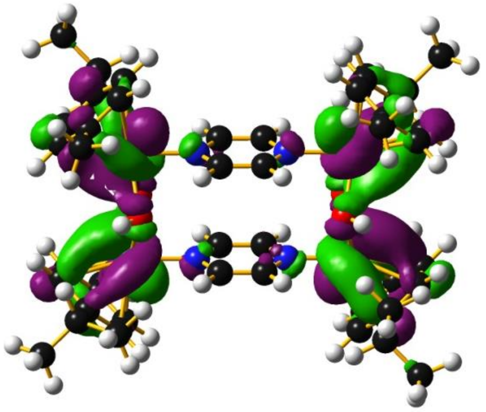 HOMO	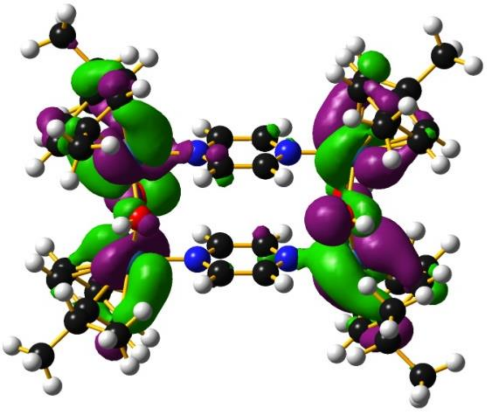 HOMO−1
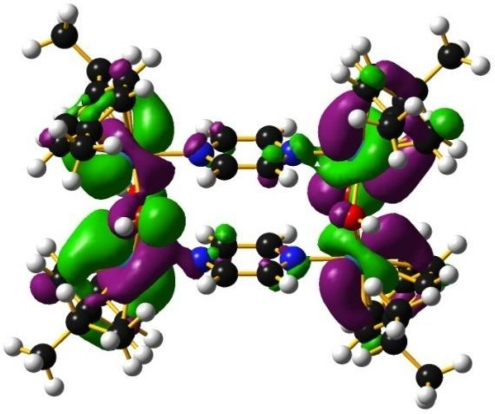 HOMO−3	

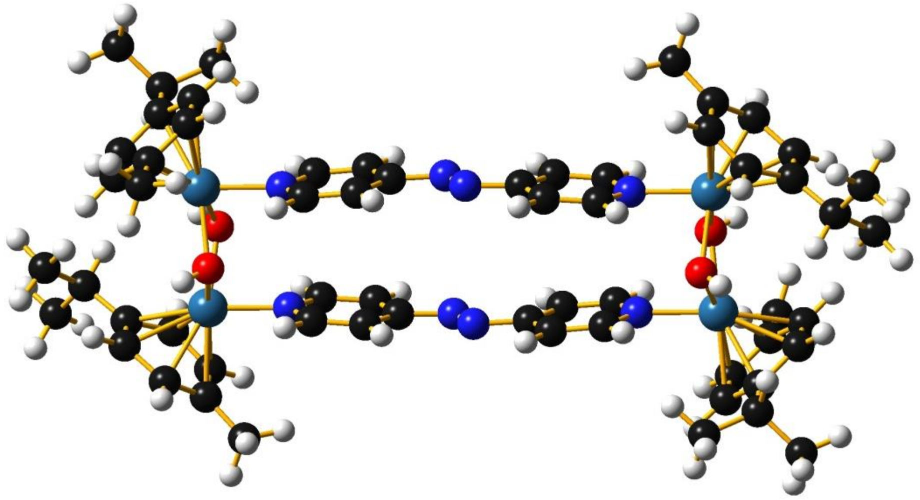

Table S6. Selected TDDFT singlet transitions for **1**·[PF_6]$_4$ in acetone.

	Energy (eV)	Wavelength (nm)	Oscillator Strength	Major contributions
1	1.77	700.49	0.0	HOMO→LUMO (96%)
2	1.7829	695.4	0.0352	H-1→LUMO (94%)
3	1.9455	637.3	0.3682	H-2→LUMO (89%)
4	1.9849	624.63	0.0	H-3→LUMO (78%)
5	2.0196	613.9	0.0007	H-13→L+1 (22%)<br>H-12→LUMO (50%)
6	2.0222	613.11	0.0659	H-13→LUMO (24%)<br>H-12→L+1 (13%)<br>HOMO→L+1 (42%)
7	2.0343	609.47	0.0	H-1→L+1 (82%)
8	2.0428	606.94	0.0324	H-13→LUMO (29%)<br>H-12→L+1 (15%)<br>HOMO→L+1 (46%)
9	2.056	603.04	0.0959	H-5→LUMO (20%)<br>H-4→LUMO (70%)
12	2.2452	552.22	0.0998	H-3→L+1 (90%)
18	2.4979	496.35	0.2719	H-9→LUMO (88%)
21	2.6616	465.83	0.1297	H-8→L+1 (26%)<br>H-6→L+1 (61%)
23	2.6901	460.89	0.3837	H-8→L+1 (64%)<br>H-6→L+1 (28%)
58	3.9184	316.42	0.1499	H-17→LUMO (67%)<br>H-16→L+1 (26%)
66	4.007	309.42	0.2289	H-3→L+4 (68%)
67	4.0528	305.92	0.4455	H-1→L+5 (10%)<br>H-1→L+6 (24%)<br>HOMO→L+7 (27%)

Table S7. Selected Electron Difference Density Maps (EDDMS) of singlet excited state transitions of **1**·$[PF_6]_4$ in acetone (pink indicates a decrease in electron density, while red indicates an increase).

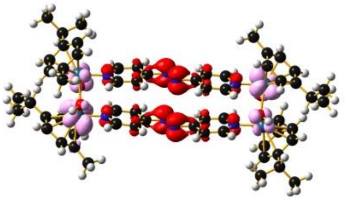 S2	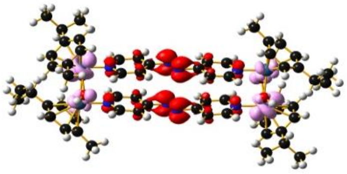 S3	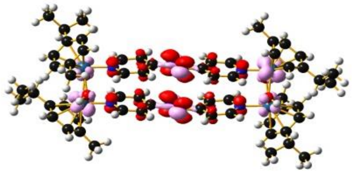 S6
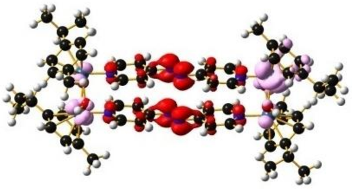 S9	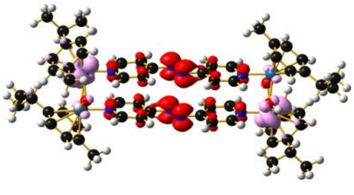 S12	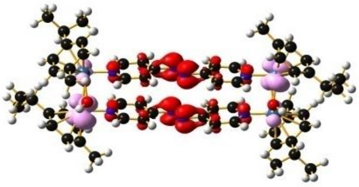 S18
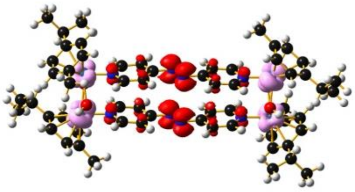 S21	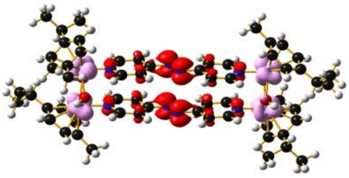 S23	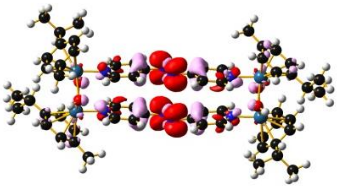 S58
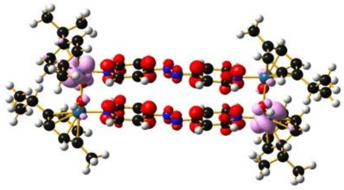 S66	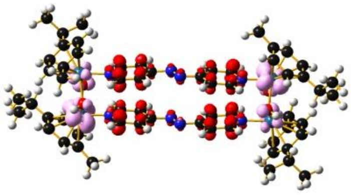 S67	

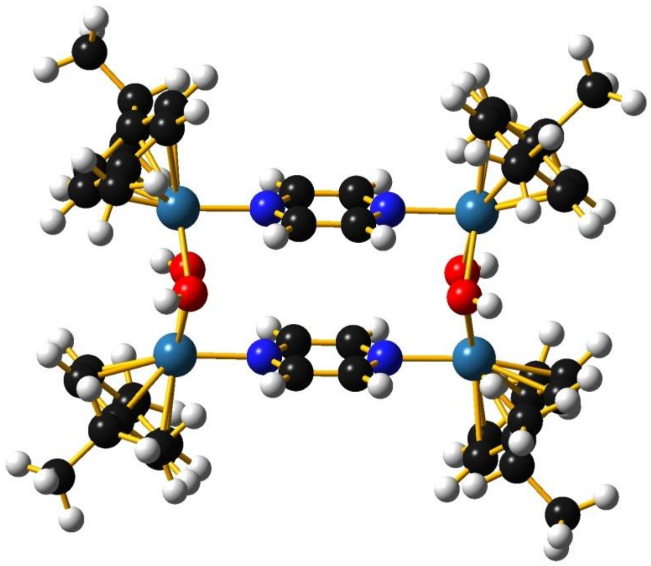

Table S8. Selected TDDFT singlet transitions for **2**·[PF_6]$_4$ in acetone.

	Energy (eV)	Wavelength (nm)	Oscillator Strength	Major contributions
1	2.25	551.04	0.0079	H-3→LUMO (13%)<br>H-1→LUMO (76%)
2	2.2879	541.9	0.0	HOMO→LUMO (93%)
3	2.307	537.43	0.0057	H-2→LUMO (88%)
4	2.3228	533.78	0.0	H-4→LUMO (96%)
5	2.4516	505.74	0.3533	H-3→LUMO (78%)<br>H-1→LUMO (13%)
20	2.979	416.2	0.2055	H-9→LUMO (73%)<br>HOMO→L+1 (14%)
33	3.4029	364.35	0.2793	H-7→L+1 (83%)
49	3.798	326.45	0.1349	H-4→L+3 (16%)<br>H-2→L+4 (21%)<br>H-1→L+4 (36%)

Table S9. Selected Electron Difference Density Maps (EDDMS) of singlet excited state transitions of **2**·$[PF_6]_4$ in acetone (pink indicates a decrease in electron density, while red indicates an increase).

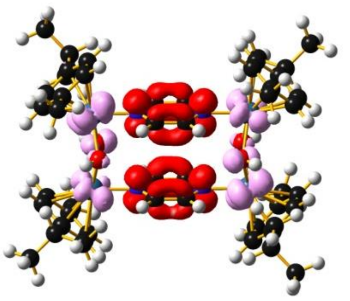 S1	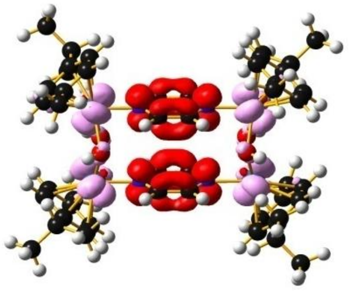 S3	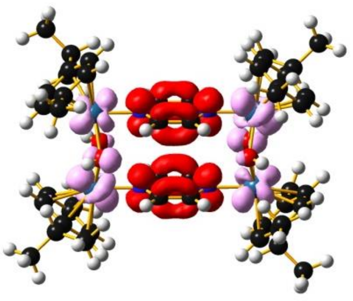 S5
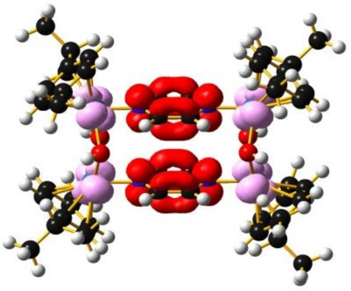 S20	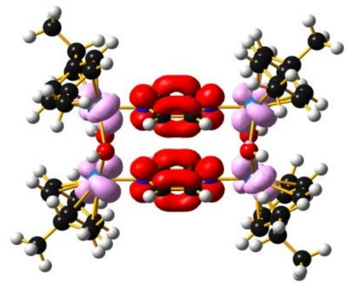 S33	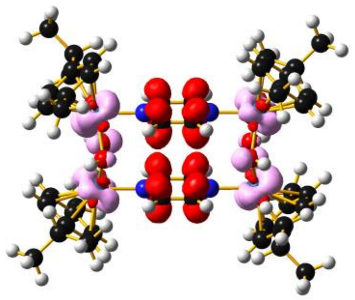 S49

(A)

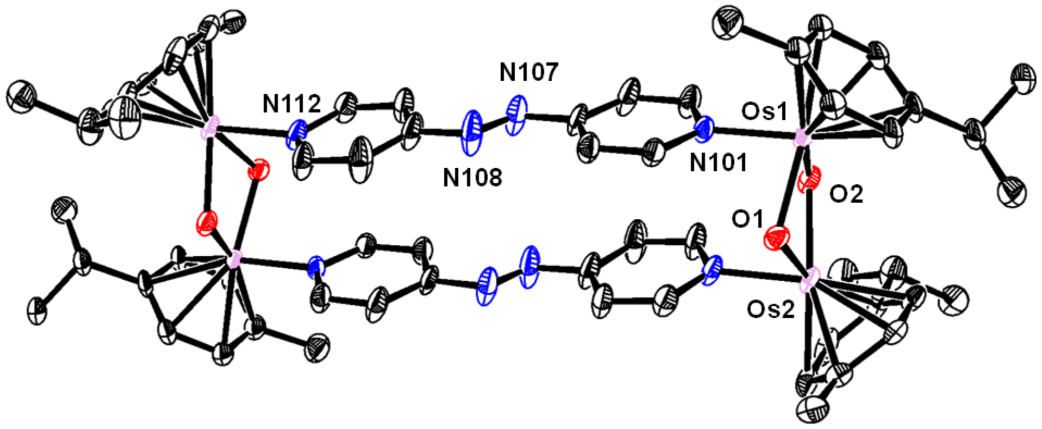

(B)

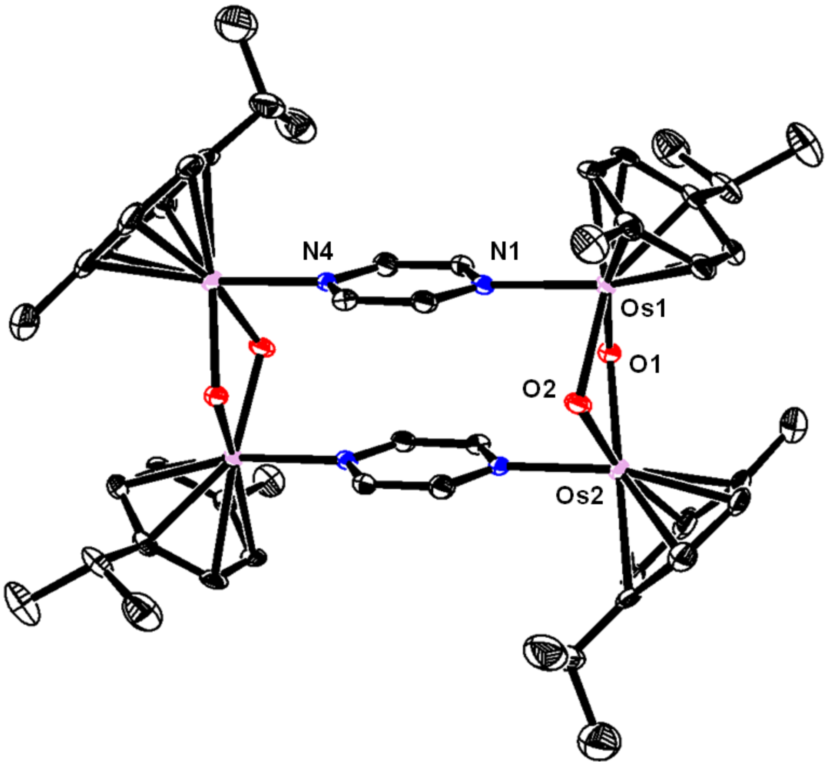

Figure S1: ORTEP diagrams of the cationic complexes (A) $[Os_4(\eta^6\text{-}p\text{-cym})_4(\mu^2\text{-}OH)_4(pap)_2]^{4+}$ **1** and (B) $[Os_4(\eta^6\text{-}p\text{-cym})_4(\mu^2\text{-}OH)_4(prz)_2]^{4+}$ **2** showing the numbering scheme. Solvent molecules, hydrogen atoms, counter ions and disorder in the isopropyl chain of *p*-cymene in complex **1** have been omitted for clarity.

The tetrameric structures of $[Os_4(\eta^6\text{-}p\text{-cym})_4(\mu^2\text{-}OH)_4(pap)_2]^{4+}$ **1**·and $[Os_4(\eta^6\text{-}p\text{-cym})_4(\mu^2\text{-}OH)_4(prz)_2]^{4+}$·**2**·each show Os^{II} coordinated to a *para*-cymene ligand through η^6 bonding, one N donor from the bridging ligand (pap or prz) and two O-donor atoms from bridging hydroxido groups. The Os−OH bond distances for both **1** and **2** (2.087(2)−2.114(4) Å) are similar to those found in other hydroxido-Os^{II} complexes (Table S2). The hydroxido-bridged Os−Os bond distances of 3.334 Å in **1** and 3.309 Å in **2** (Table S2), are longer than those reported for hydroxido-bridged Os^{II} benzene complexes.[20]

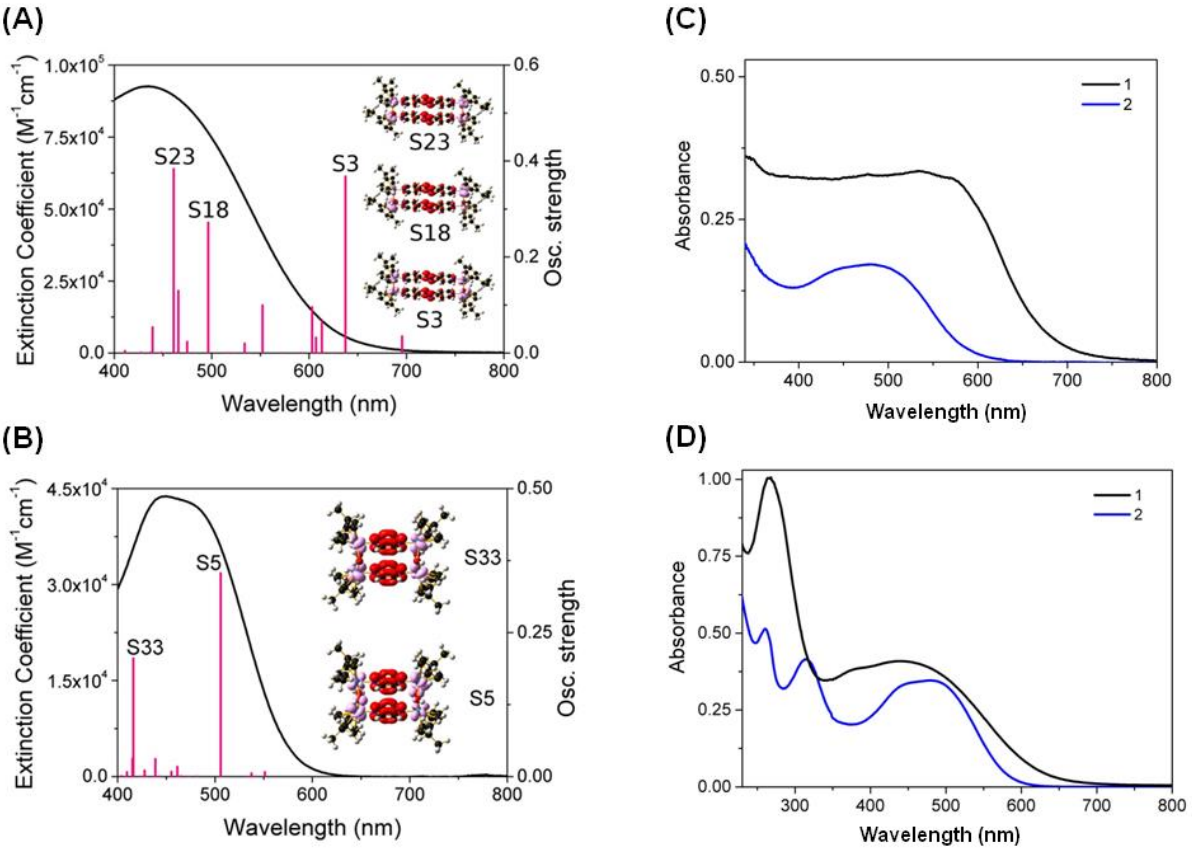

Figure S2. Experimental absorption spectrum (black line) and calculated singlet electronic transitions (S, vertical bars) for (A) **1**·$[PF_6]_4$, and (B) **2**·$[PF_6]_4$ in acetone. Inset: selected electron difference density maps of singlet excited state transitions (pink indicates a decrease in electron density, while red indicates an increase). The electron density difference maps for **1** and **2** show the ^{1}MLCT nature of the bands at 435–450 nm. UV-Visible absorption spectra of **1**$[PF_6]_4$ (black) and **2**$[PF_6]_4$ (blue) (C) in acetone, and (D) in methanol.

Notes: UV-vis absorption spectra of **1**·$[PF_6]_4$ and **2**·$[PF_6]_4$ in acetone (Fig. S2C) show a broad absorption band between 400 nm and 800 nm, with an extinction coefficient of 92600 $M^{-1}cm^{-1}$ at the λ_{max} of 435 nm for complex **1**·$[PF_6]_4$ and 43800 $M^{-1}cm^{-1}$ at 450 nm for **2**·$[PF_6]_4$. The absorption profile of complex **1**·$[PF_6]_4$ is broader and the tail between 600−700 nm explains why concentrated solutions of **1**·$[PF_6]_4$ (0.75 mM) are black/brown-colored whereas solutions of **2**·$[PF_6]_4$ are red. Similar features are present in methanol (Fig. S2D) where it is also possible to observe intense absorption peaks below 300 nm, corresponding to transitions which have ligand-centered (pap and prz) and metal-centered character.

(A)

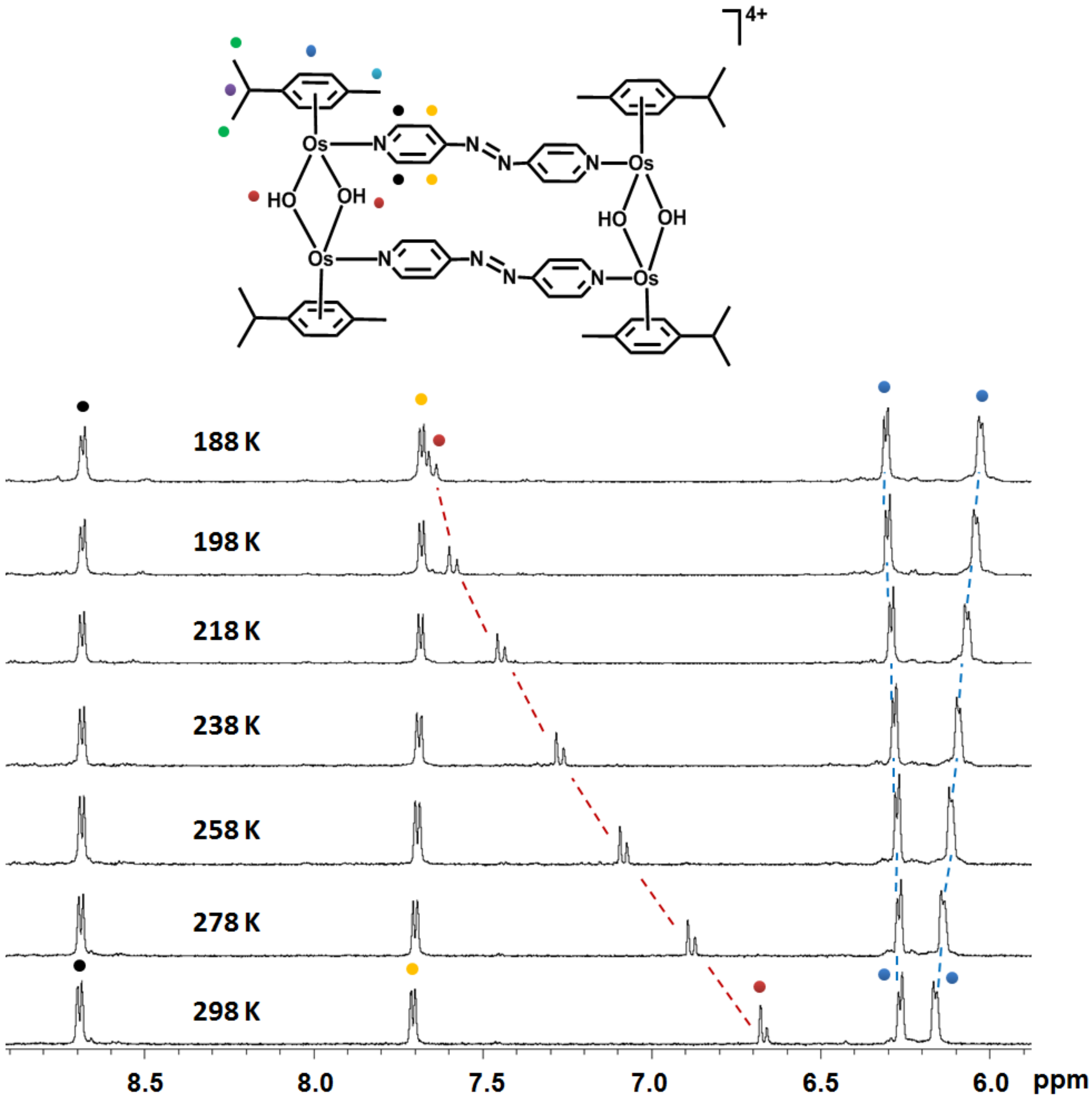
4+
Os
N
N
N
N
Os
HO
OH
Os
N
N
N
N
Os
HO
OH
188 K
198 K
218 K
238 K
258 K
278 K
298 K
8.5
8.0
7.5
7.0
6.5
6.0
ppm

(B)

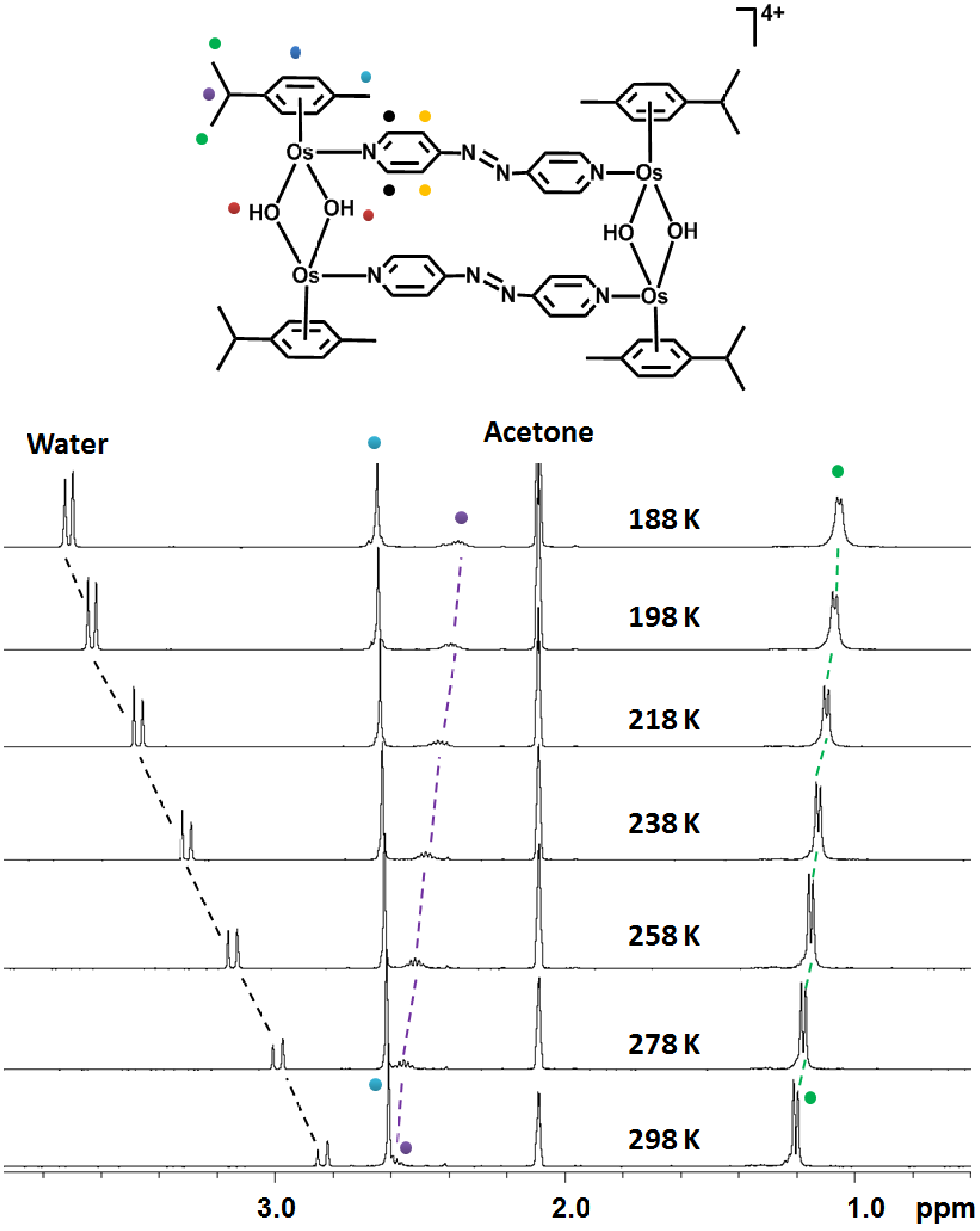

4+
Os
N
N
N
N
Os
HO
OH
Os
N
N
N
N
Os
HO
OH
Water
Acetone
188 K
198 K
218 K
238 K
258 K
278 K
298 K
3.0
2.0
1.0
ppm

(C)

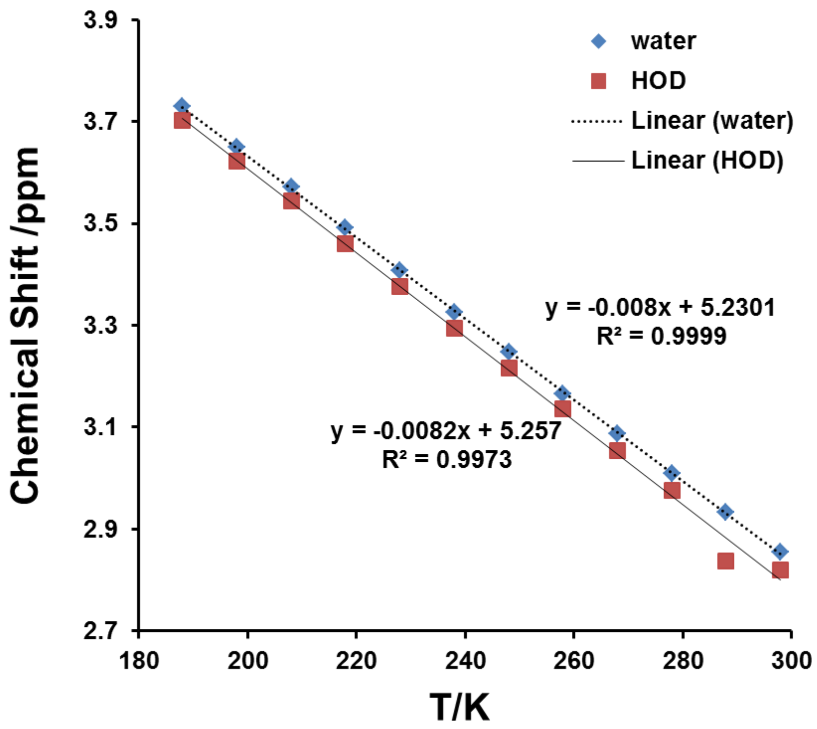

(D)

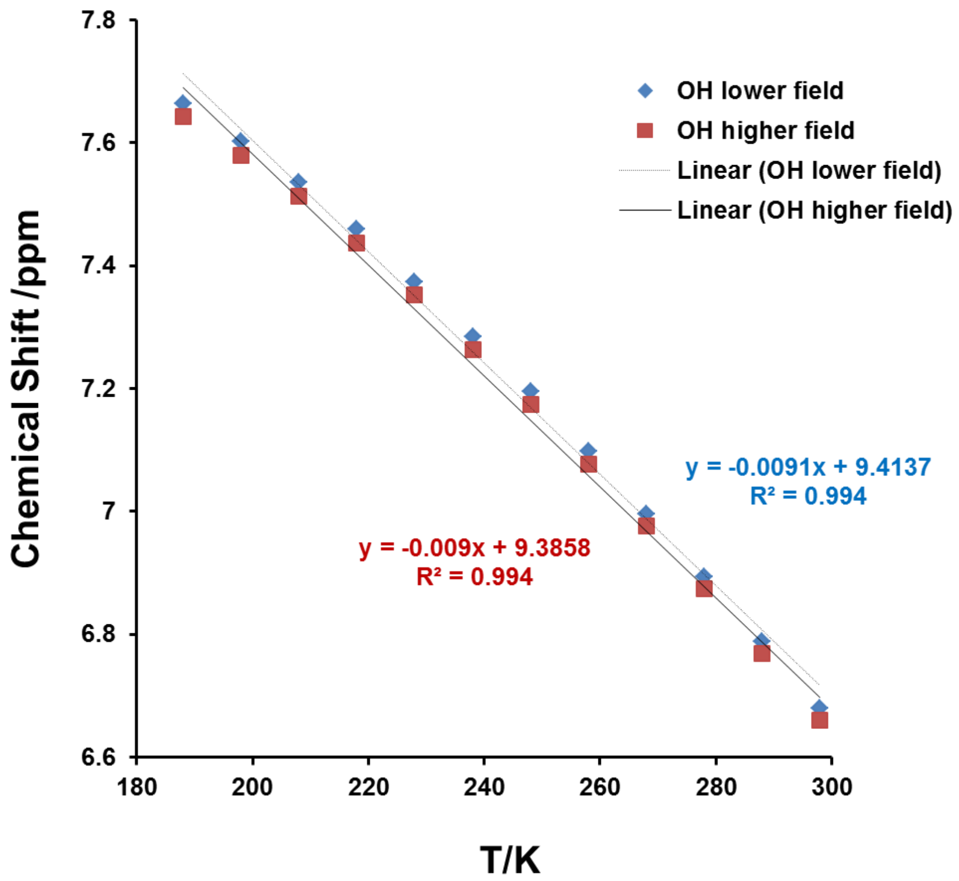

Figure S3. ^{1}H NMR spectra of **1**·$[PF_6]_4$ recorded at various temperatures (298 K to 188 K) in acetone-d_6: (A) Stacked spectra for the region 5-9 ppm; (B) Stacked spectra for the region 0.5-4 ppm; (C) Plot of chemical shift *versus* temperature for water and HOD peaks; (D) Plot of the chemical shift corresponding to OH peaks *versus* temperature.

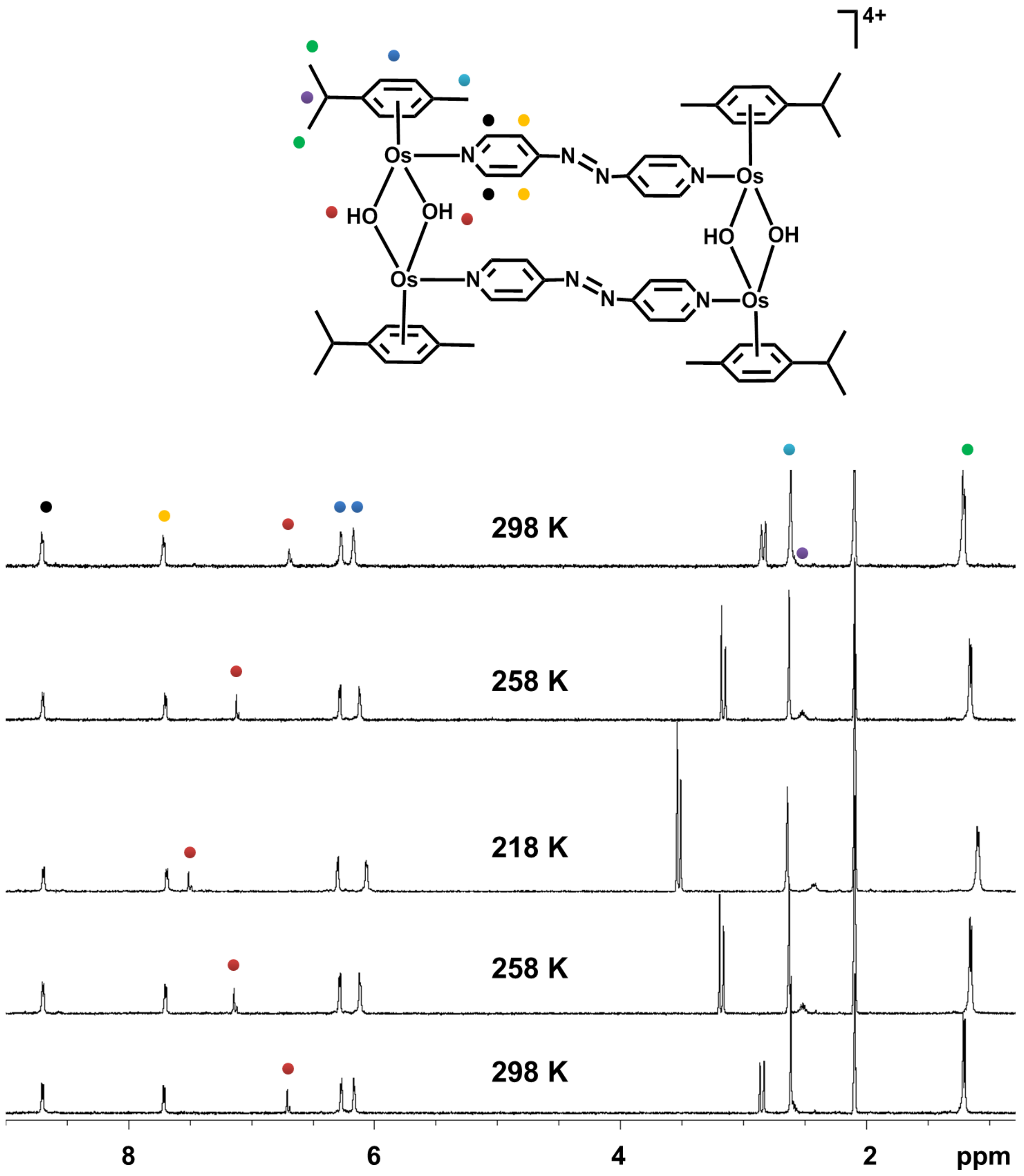

Figure S4. Dependence of the ^{1}H NMR spectrum of **1**·$[PF_6]_4$ on temperature (298 K → 258 K → 218 K with reversal to 258 K → 298 K) in acetone-d_6.

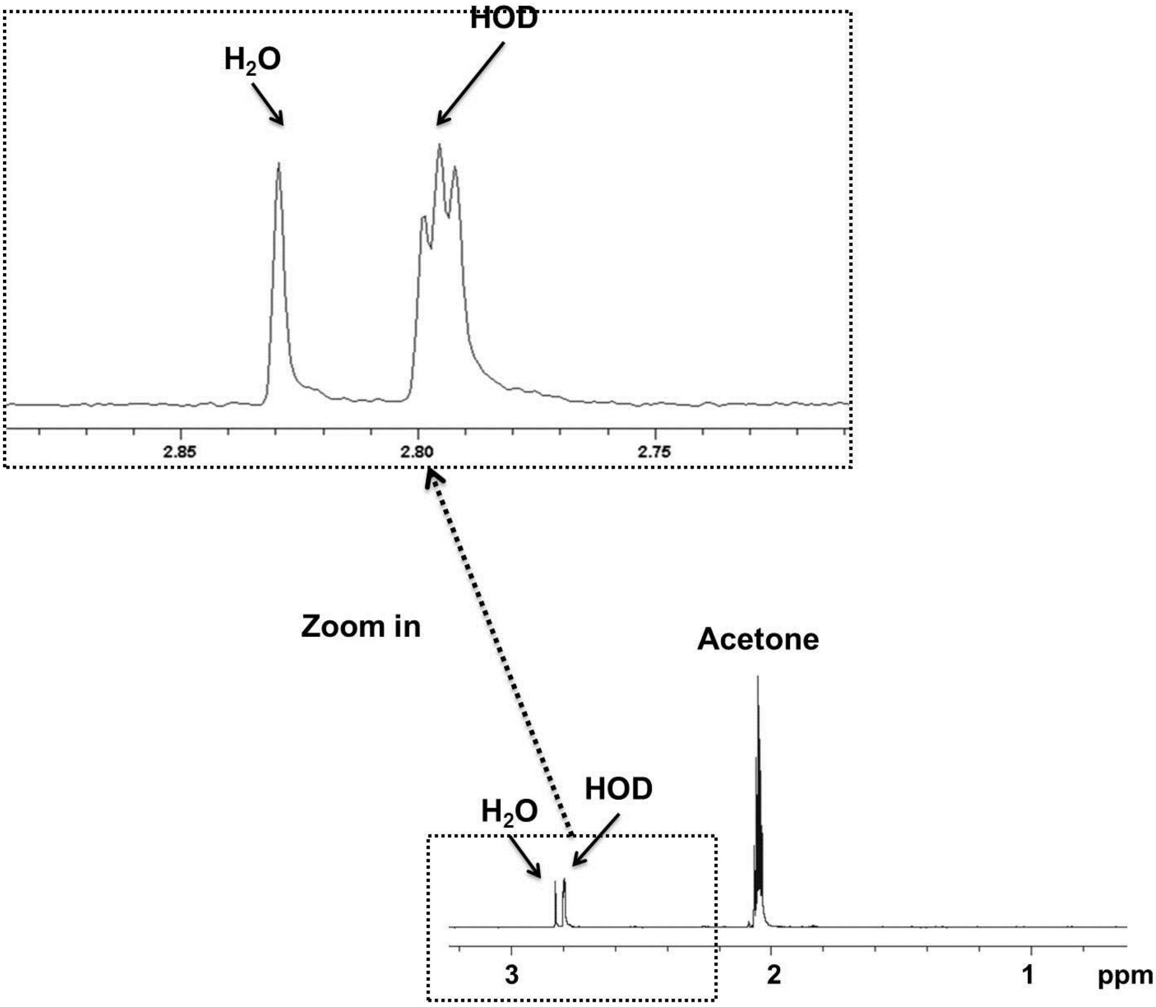

Figure S5. ^{1}H NMR spectrum of acetone-d_6 (run immediately after opening the bottle).

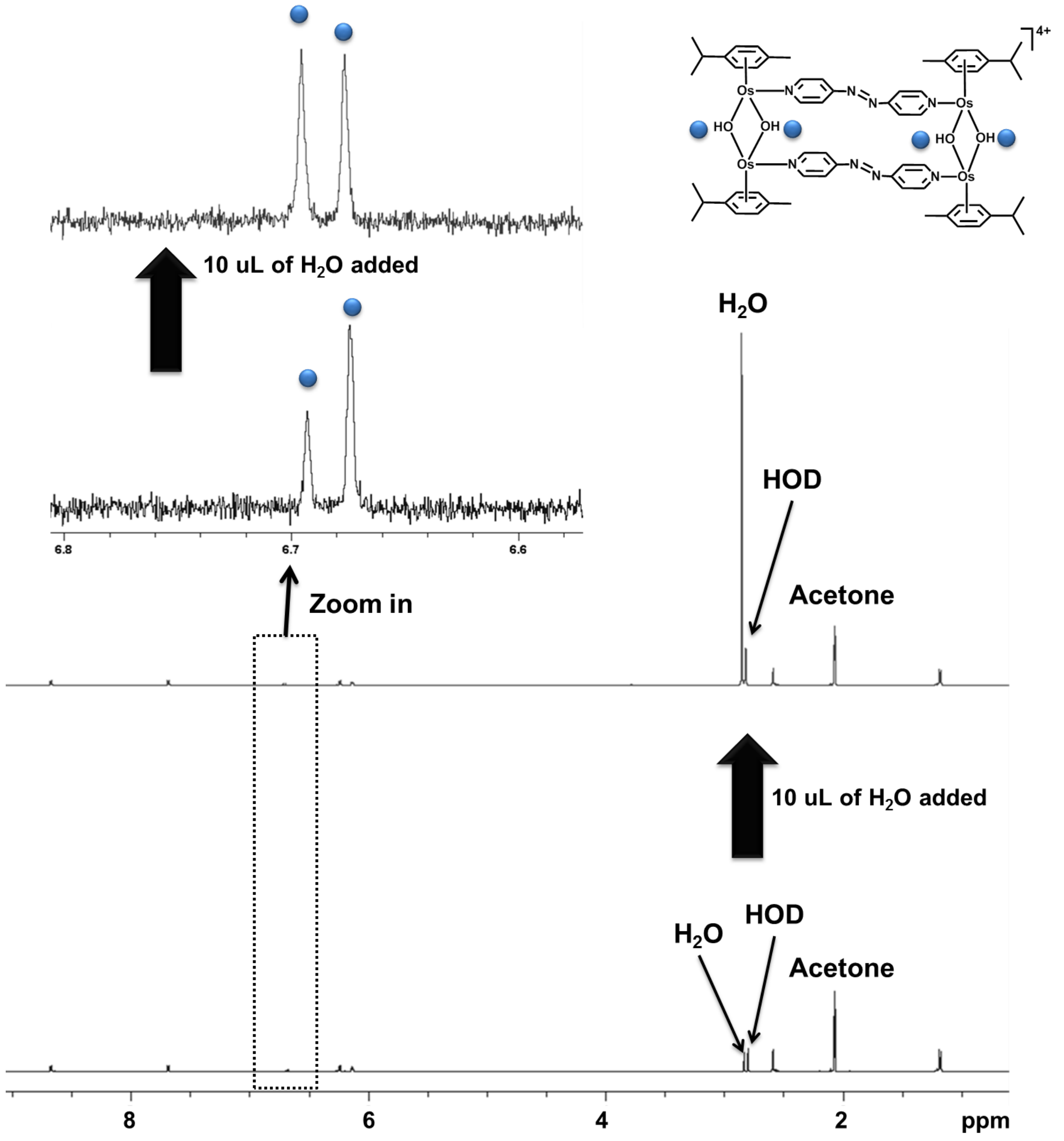

Figure S6. ^{1}H NMR spectrum of **1**·$[PF_6]_4$ (600 µL, 1 mM in acetone-d_6) before and after addition of 10 µL of H_2O. The inset shows the increase of intensity for the signal for one OH bridge in the tetracationic complex **1**.

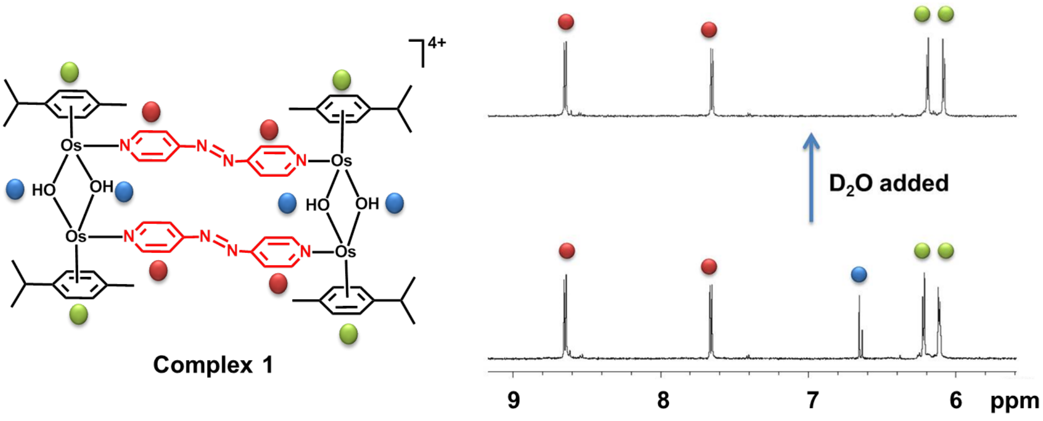

Figure S7. ^{1}H NMR spectrum of **1**·$[PF_6]_4$ in acetone-d_6 before and after 10 µL of D_2O was added to the sample.

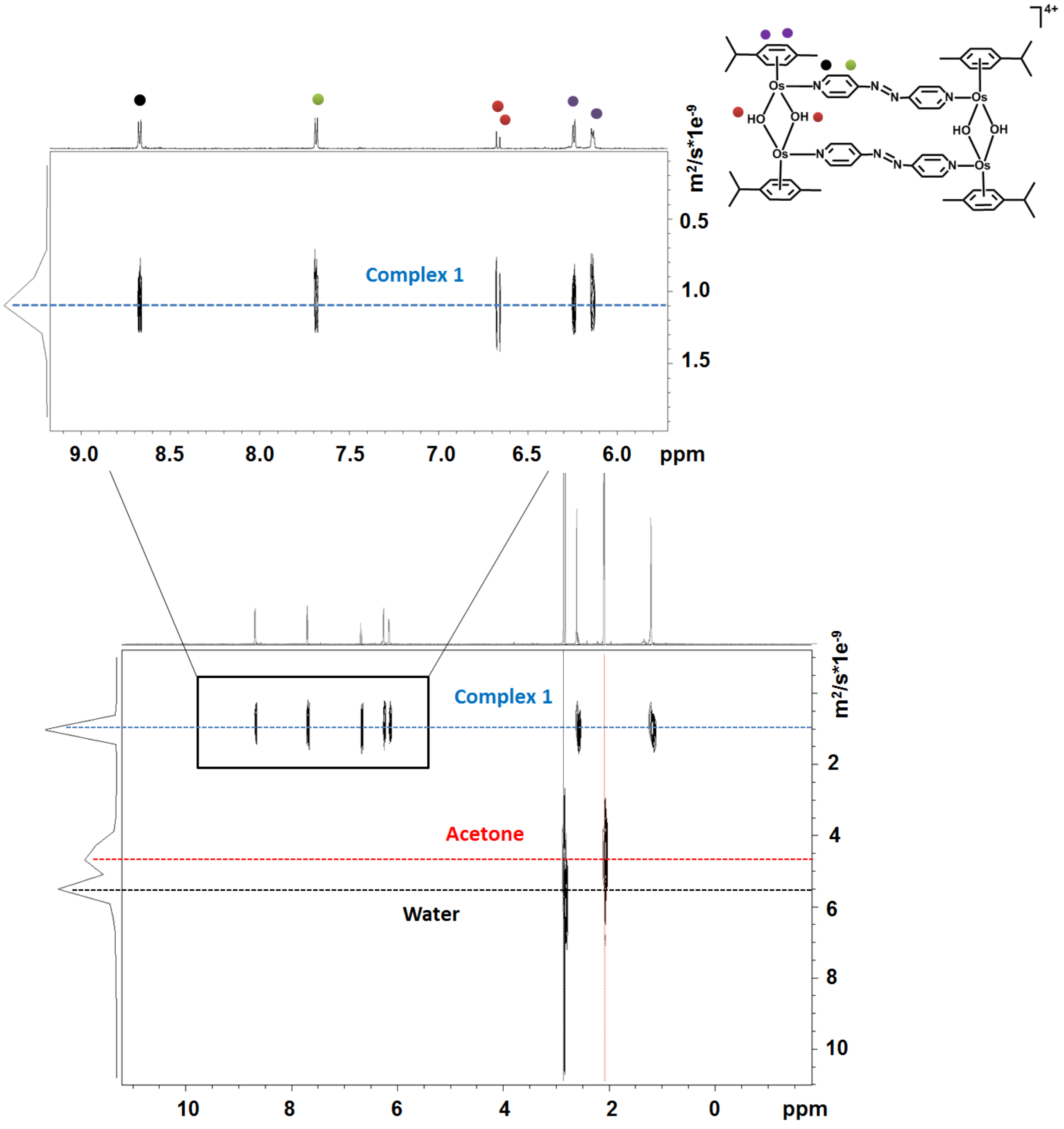

Figure S8: ^{1}H NMR DOSY spectrum for tetramer **1**·[PF$_6$]$_4$ (600 µL, 1 mM) in acetone-d$_6$. The y axis corresponds to the diffusion coefficient.

(A)

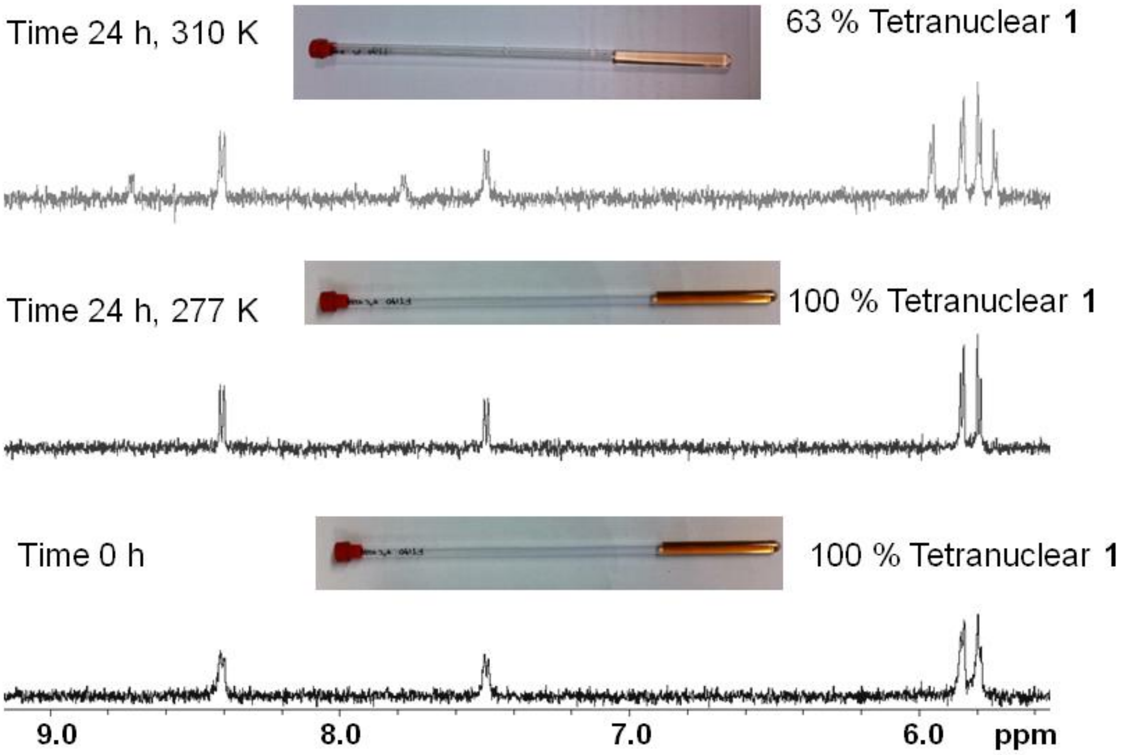
Time 24 h, 310 K
63 % Tetranuclear 1
Time 24 h, 277 K
100 % Tetranuclear 1
Time 0 h
100 % Tetranuclear 1
9.0
8.0
7.0
6.0
ppm

(B)

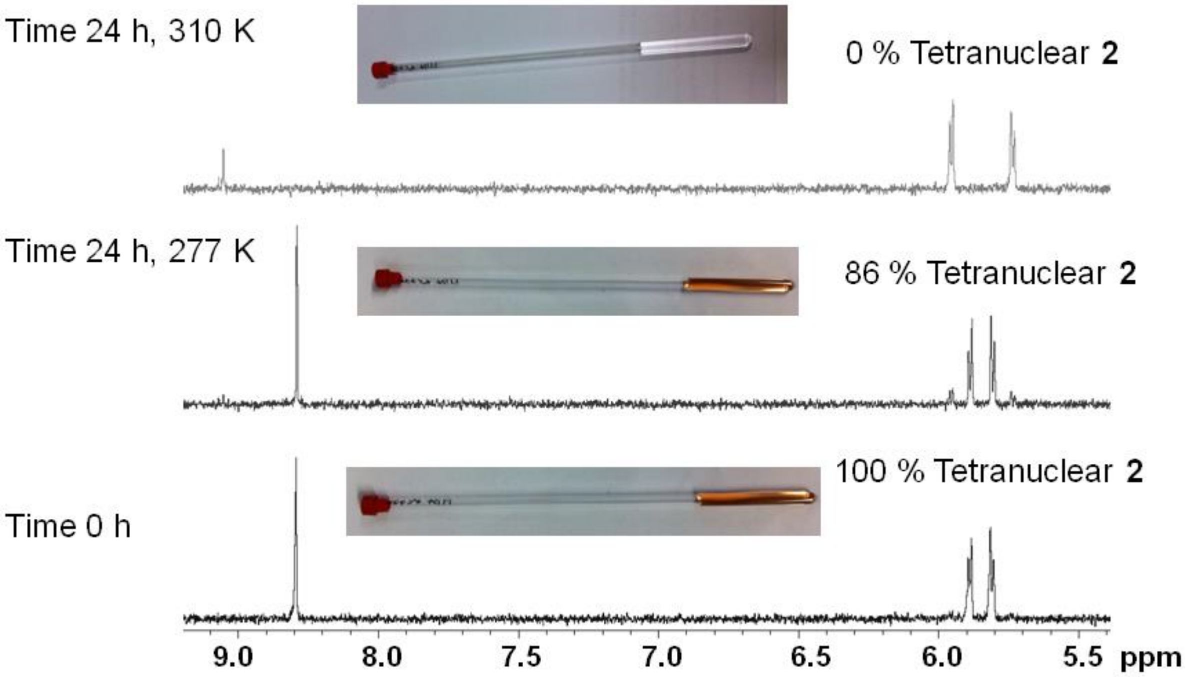
Time 24 h, 310 K
0 % Tetranuclear 2
Time 24 h, 277 K
86 % Tetranuclear 2
100 % Tetranuclear 2
Time 0 h
9.0
8.0
7.5
7.0
6.5
6.0
5.5
ppm

(C)

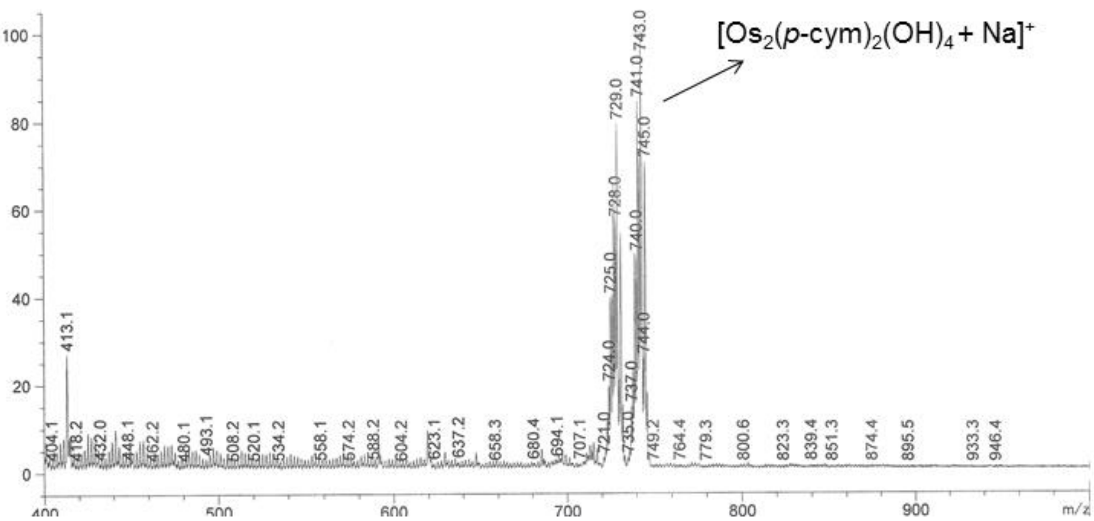

(D)

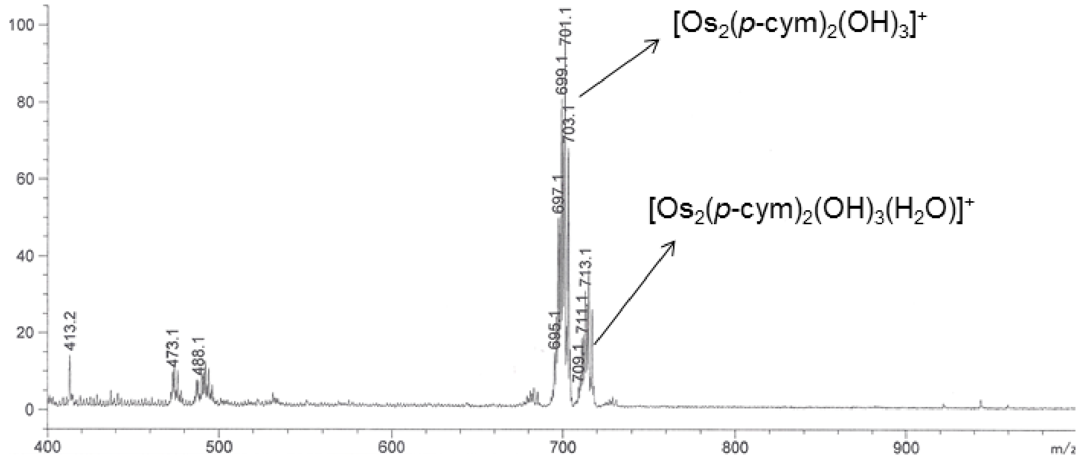

Figure S9. ^{1}H NMR spectra of the tetranuclear complexes **1**·$[PF_6]_4$ (A) and **2**·$[PF_6]_4$ (B) in 10%/90% MeOD-d_4/ D_2O phosphate buffer (1 mM, pH^*= 7.4) before and after incubation at various temperatures for 24 h. Peaks assigned to the new species ($[Os_2(p\text{-cym})_2(OH)_4\text{+}Na]^+$, 743.0 m/z and $[Os_2(p\text{-cym})_2(OH)_3]^+$, 701.1 m/z) were detected by ESI$^+$-MS for tetramer **1**·$[PF_6]_4$ (C) and tetramer **2**·$[PF_6]_4$ (D) from aqueous solutions incubated for 24 h at 310 K.

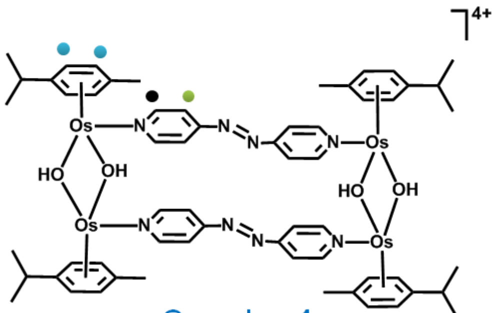

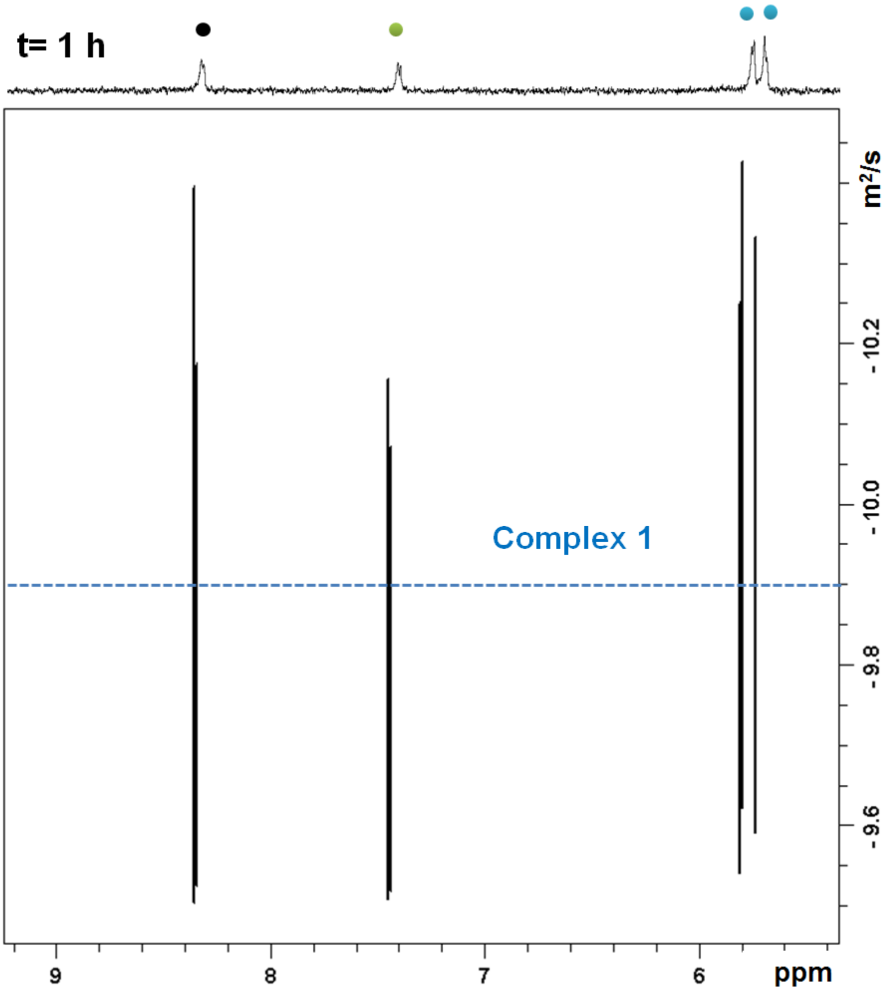

Figure S10: 2D DOSY ^{1}H NMR spectrum of **1**·$[PF_6]_4$ recorded at t= 1 h after dissolution of the complex in 10% MeOD-d_4/90% D_2O sodium cacodylate buffer (1 mM, pH* 7.4). No new species were detected at this time point. The y axis corresponds to the diffusion coefficient (m^2/s).

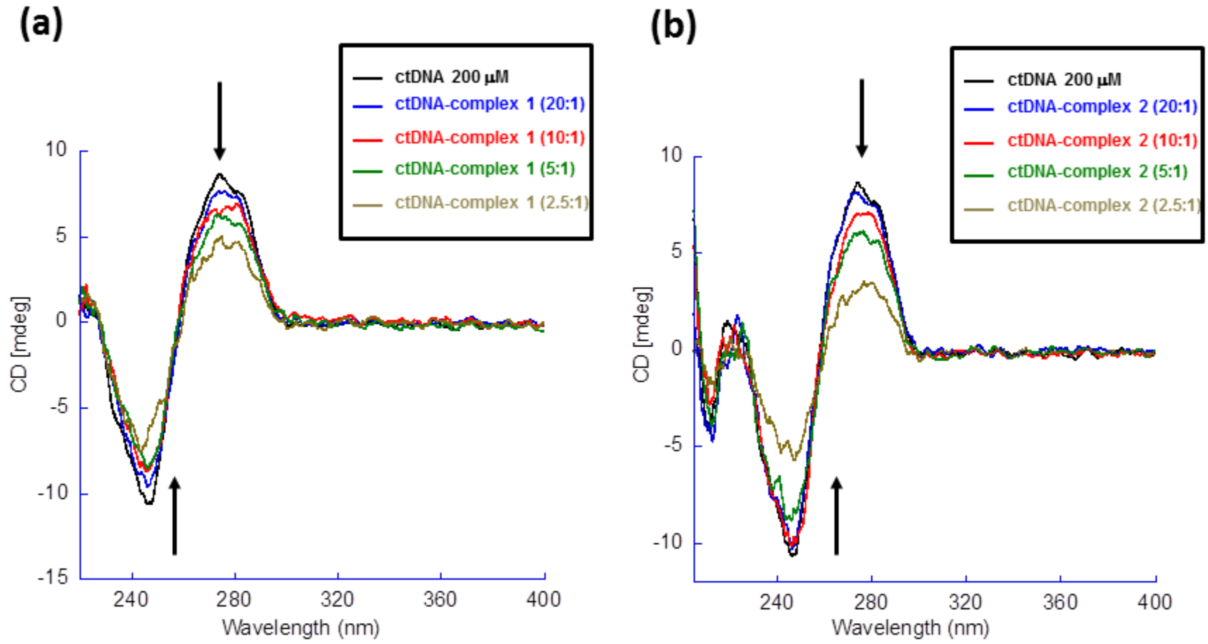

Figure S11. Circular dichroism (CD) titration of tetramers **1**$[PF_6]_4$ (a) and **2**$[PF_6]_4$ (b) with ct-DNA (200 μM) at different mol ratios (1 mM sodium cacodylate buffer, 20 mM NaCl, pH 7.2). The molar ellipticity for the ct-DNA bands assigned to the nucleobase stacking (at *ca.* 280 nm) and B-DNA helicity (at *ca.* 250 nm) decreased in intensity upon increasing the Os_4:DNA base mol ratio. No induced CD bands in the visible region were observed in these experiments, possibly because they are very weak.

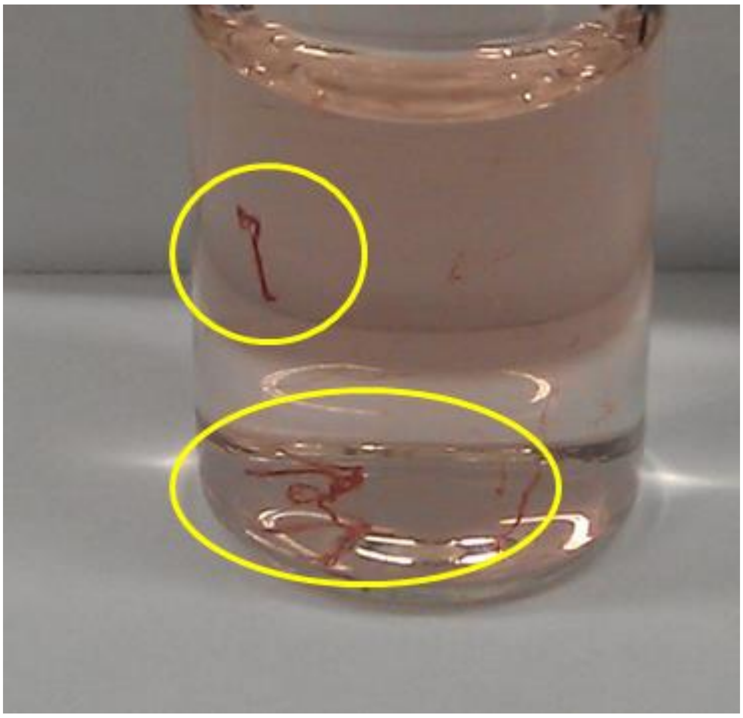

Figure S12. The addition of **2**·$[PF_6]_4$ (400 μM in 5% DMSO/95% sodium cacodylate buffer) to a ct-DNA solution (200 μM in 1 mM sodium cacodylate buffer, 20 mM NaCl, pH 7.2) at a Os_4:DNA base mol ratio >1:2 induced the precipitation of red fibers corresponding to a ct-DNA-Os(II) tetramer adduct.

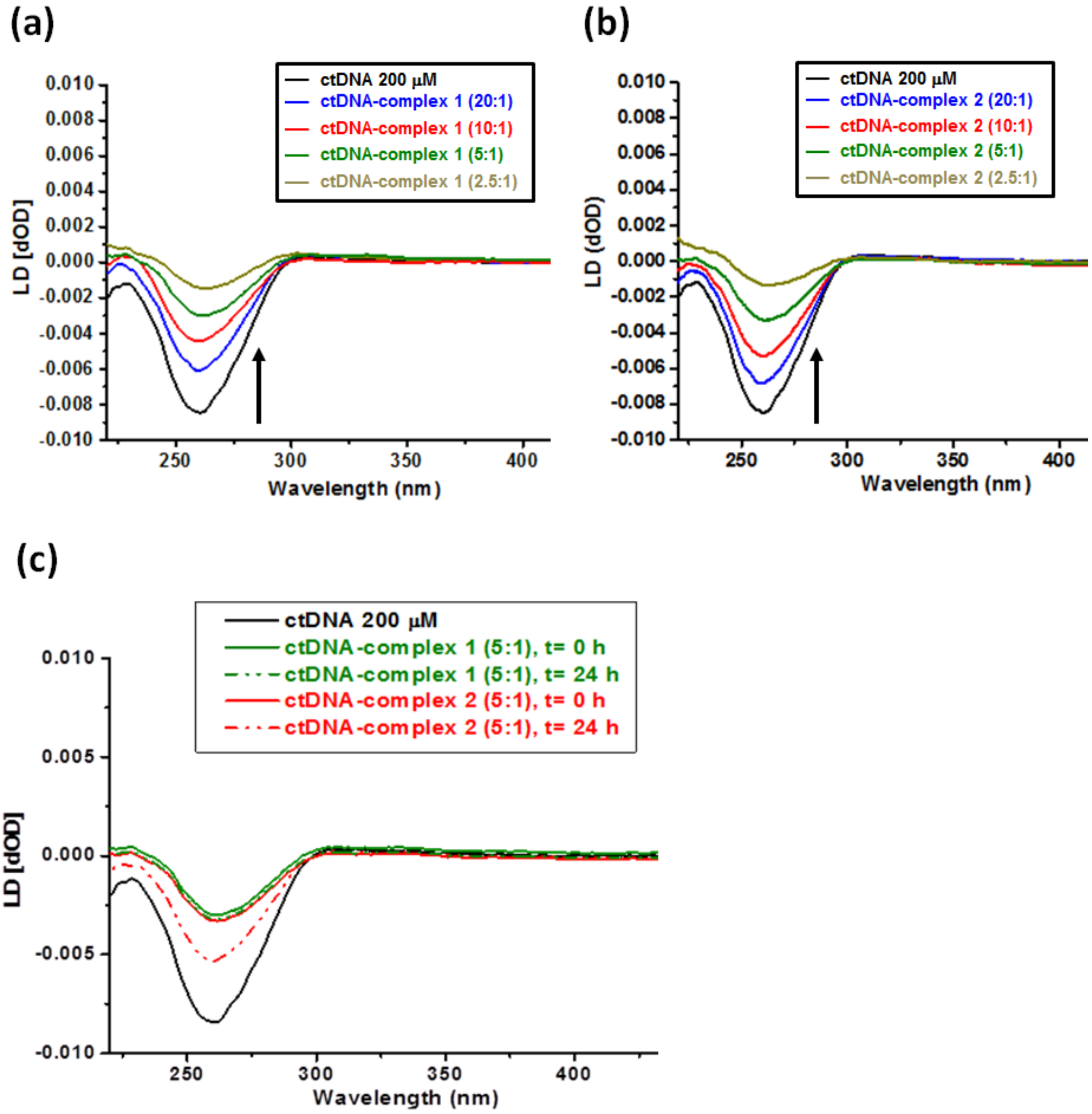

Figure S13: Linear dichroism (LD) spectra recorded for solutions of ct-DNA with the tetramers **1**· $[PF_6]_4$ (a) and **2**· $[PF_6]_4$ (b) at different DNA base:tetramer ratios. Comparison of LD spectra (c) for a ratio 5:1 at t = 0 h and t = 24 h after incubating the mixture at 310 K (1 mM sodium cacodylate buffer, 20 mM NaCl, pH 7.2).

Notes: The intensity of the negative band (ca. 260 nm) remained stable in the case of the DNA-**1** adducts after incubation at 310 K for 24 h, however this was not the case for the adducts with complex **2**.

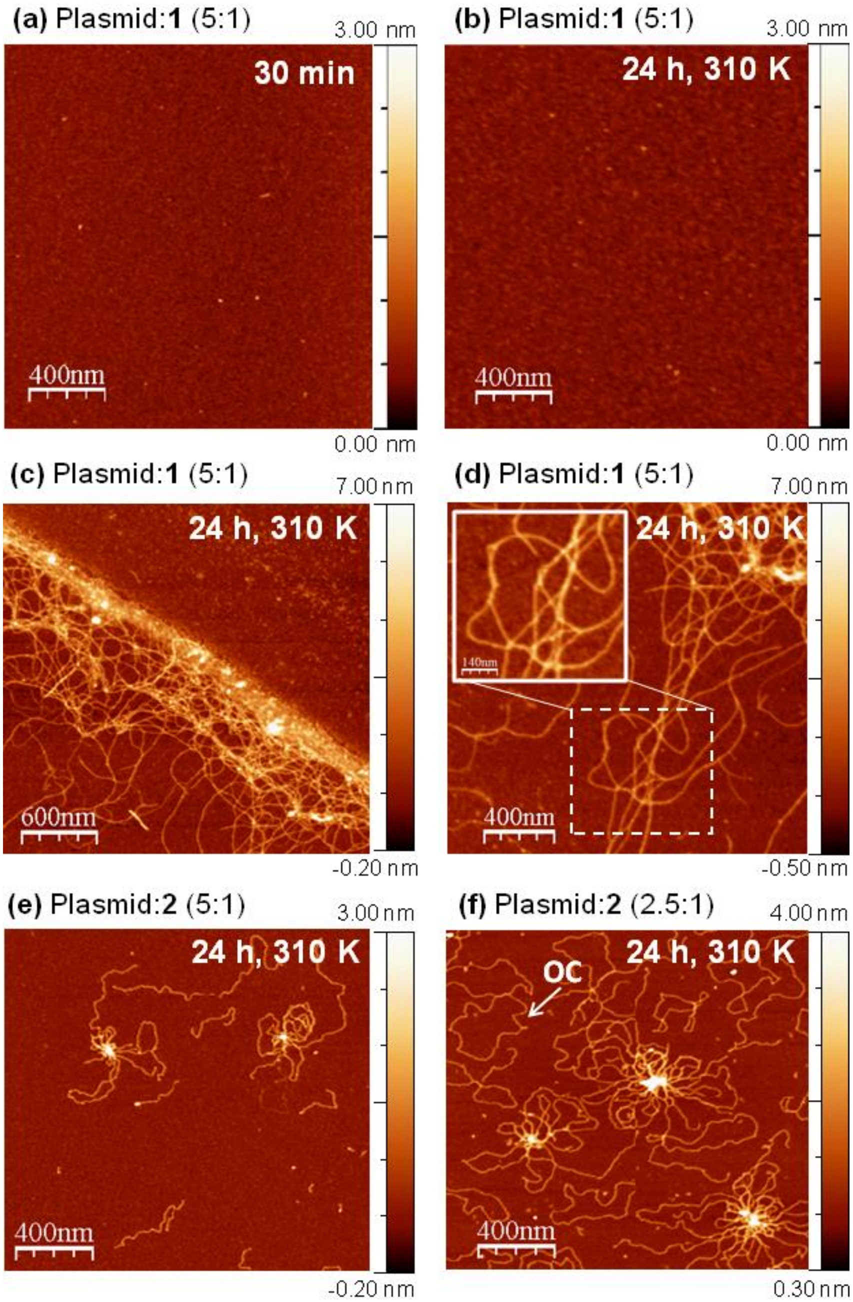

Figure S14: (a-d) AFM images for pBR322 plasmid DNA in the presence of tetramer **1**$[PF_6]_4$ in a DNA base:tetramer ratio of 5:1. (a, b) No plasmids were detected on the mica surface. Images (a, b) were recorded 30 min after preparing the sample and 24 h after incubation at 310 K, respectively. (c, d) These images correspond to a very rare case where a large network of cross-linked plasmids could be observed close to a surface step edge while the remaining part of the sample did not show any plasmid. DNA cross-links are occasionally observed as bright protrusions with a height of 3.2 ± 0.5 nm (n=12) along the DNA strands. (e, f) Comparison of AFM images for pBR322 plasmid DNA in the presence of complex **2**$[PF_6]_4$ at DNA base: tetramer mol ratios of 5:1 (e) and 2.5:1 (f), respectively. The density of the free OC plasmids in (e, f) is not homogeneous over the surface. The images (c-f) were recorded after incubating the samples for 24 h at 310 K. A color-height scale is shown on the right. Conditions: 2 mM HEPES buffer, pH 7.2, 0.9 mM $MgCl_2$, plasmid concentration in solution 1.6 μg/mL.

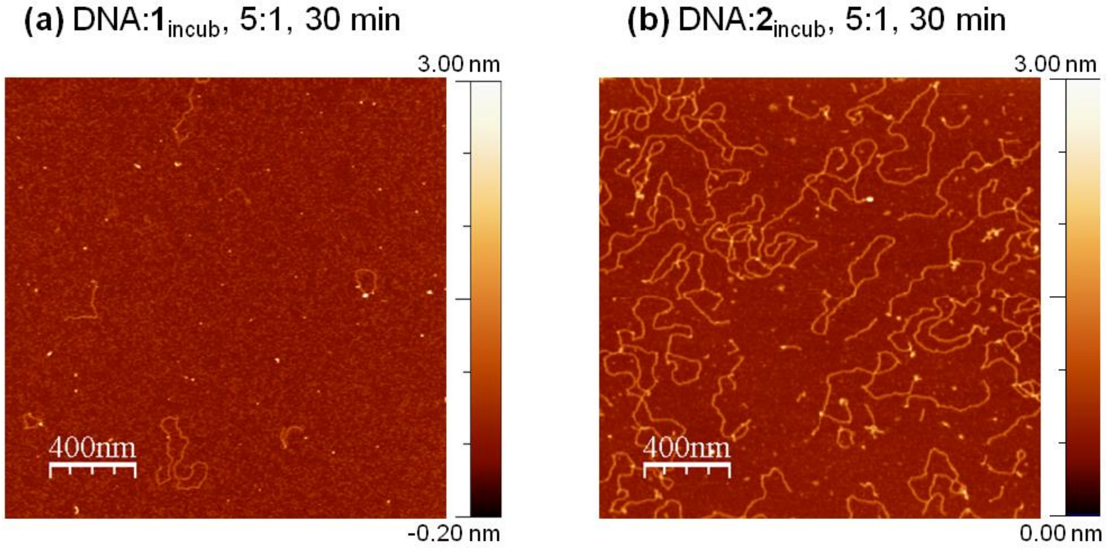

Figure S15: AFM images of pBR322 plasmid DNA in the presence of the pre-incubated (24 h, 310 K) tetramers **1**·$[PF_6]_4$ and **2**·$[PF_6]_4$ at a DNA base: tetramer mol ratio 5:1. The images were recorded at t = 30 min after preparing the samples. A color-height scale is shown on the right of each image. Conditions: 2 mM HEPES buffer, pH 7.2, 0.9 mM $MgCl_2$, plasmid concentration in solution 1.6 μg/mL.

The interaction of metallosupramolecular complexes with DNA, including those derived from ruthenium[21] and rhodium[22], has been previously studied by other authors using different techniques. However studies on organometallic osmium complexes using AFM are scarce. Our AFM experiments and discussion of results are detailed in the following sections.

Discussion of AFM results:

1. AFM analysis of free pBR322 plasmid DNA: AFM images were recorded 30 min after mixing and again after 24 h of incubation at 310 K. Typical images of the control sample where the free pBR322 plasmid was deposited onto Mg^{2+}-treated freshly-cleaved mica (needed for a firm adsorption of the negatively charged DNA) are shown in Figure 3 (main script). Mainly relaxed open circular (OC) and linear (L) forms were observed on the surface. Analysis of cross sections of the different forms in the control sample resulted in heights of 0.70 ± 0.08 nm (OC) and 0.7 ± 0.1 nm (L), respectively (mean value ± standard deviation; number of samples n=16). A few bright dots also observed on the mica substrate were identified as buffer salt deposits (height 1.3 ± 0.3 nm, n=16). The measured DNA height is smaller than the theoretical DNA diameter (ca. 2 nm), probably due to the effect of the electrostatic interactions with the surface, as previously suggested by other authors.[23] Reported apparent heights of double-stranded DNA deposited onto mica vary within the range 0.5-2.0 nm (AFM operating in air).[24] Images of the free plasmid were recorded as controls for all the samples under the same experimental conditions; these confirmed the stability of the plasmid after 24 h of incubation at 310 K.

2. AFM analysis of the interaction of plasmid DNA with tetramer 1: Topographical images for the interaction between cationic complexes **1** and **2** with pBR322 plasmid DNA (4363 bp) are shown in Figure S14 and Figure 3 (main script). The Os^{II} complexes were added to solutions of pBR322 DNA plasmid at a DNA base: Os_4 ratio of 5:1.The image recorded 30 min after mixing pBR322 plasmid with tetramer **1**·$[PF_6]_4$ (the more biologically-active complex) resulted in only a few particles of buffer salts observed on the mica surface (Fig. S14a) with an average height of 1.4 ± 0.5 nm (n=22), comparable to those observed in the control samples (Figs. 3a, b, main script). However, no plasmids could be detected on any of the analyzed samples. These results indicate that the DNA adduct formed by the OC or L plasmids with complex **1** (4+ charge) was less negatively charged than the pristine DNA, hence could not readily adsorb onto the positively-charged substrate and thus could not be observed by AFM. The incubation of the solution of the DNA: **1** adduct for 24 h at 310 K gave rise to an identical situation in which only buffer salt particles were observed (Fig. S14b) with a height (1.7 ± 0.3 nm; n=22) similar to that of the particles before and after the incubation period of 24 h (Figs. 3a, b, main script).

Surprisingly, it was however possible to image a large network of cross-linked plasmids close to a step edge of the surface on the same sample (Figs. S14c, d). The network appeared to be directly anchored to the step and it was not possible to find any other similar structure

on the terraces of the Mg^{2+}-treated mica surface. Such a special situation might be due to a different termination or surface charge density of the step with respect to the planar regions of the substrate. The analysis of segments of the DNA strands involved in the network (Fig. S14c, d) resulted in an average height of 2.2 ± 0.3 nm (n=26), which is ca. three times larger than the value observed for the OC and L forms of the free plasmid (Figs. 3a, b, main script). This is most probably due to the binding of the positively-charged tetramer **1** to the pBR322 DNA plasmid which reduces its electrostatic binding to the substrate and thus increases its height when measured by AFM.

3. AFM analysis of the interaction of plasmid DNA with tetramer 2: The images of the pBR322 plasmid DNA changed drastically upon addition of the less biologically-active complex **2**·$[PF_6]_4$. Interaction with tetramer **2**·$[PF_6]_4$ gave rise to the formation of aggregates of plasmid DNA which did stick onto the substrate and appeared as plasmid loops (maximum loop length along the longitudinal DNA axis of ca. 400 nm) emerging from a condensed nucleus, probably generated by the linking of several DNA strands mediated by complex **2** (see Fig. 3c, main script). The height of the plasmid DNA loops was 1.2 ± 0.2 nm (n=19), i.e. larger than the free plasmid but smaller than the DNA: **1** adducts described in the previous section, while the condensed nuclei displayed a height of 5.8 ± 1.2 nm (n=7). Additionally, plasmid molecules with an OC and L structure were observed on the surface (Fig. 3c, main script) with a height of only 0.70 ± 0.06 nm (n=14). These latter molecules were assigned to unreacted free DNA plasmids.

The lateral extension of the plasmid loops became larger and the loops length along the DNA axis increased to values ranging from 600 to 900 nm after incubating the sample for 24 h at 310 K (Fig. 3d, main script). This change can be associated with the cleavage of the tetranuclear structure **2** during the incubation period (loss of bridging ligands), thus partially releasing plasmids from the central core.

The observations that, unlike the aggregates formed with complex **1**, the DNA adducts with complex **2** are strongly attached to the Mg^{2+}-treated mica and their height is smaller than that of the DNA-**1** adducts (i.e. they are subject to a stronger height reduction induced by electrostatic interactions with the substrate), both suggest a larger negative charge density of DNA-**2** with respect to DNA-**1**. Given that both complexes **1** and **2** have a 4+ charge, the difference in charge density is most probably due to the smaller number of bound molecules **2** with respect to **1**, caused by the more facile decomposition of **2**.

Experiments carried out at a lower DNA base:tetramer **2** mol ratio (2.5:1) showed the formation of similar aggregates with loops emerging from a central condensed nucleus together with OC structures of free plasmids (Fig. S14f). With respect to the 5:1 ratio, the overall surface density of the DNA aggregates increased and the height of the DNA loops was marginally larger, 1.4 ± 0.3 nm (n=18), but still comparable to what observed in Fig. 3c (main script). Both observations confirm a higher linear density of **2** bound to the DNA strands resulting in the formation of more aggregates with the same shape but a slightly increased height (Fig. S14f) when using a lower DNA base:tetramer **2** ratio. Additionally, more plasmid

molecules are attached to the central cores and the extension of some of the aggregate loops is larger (maximum length of ca. 1.4 μm along the DNA axis) than observed for the 5:1 ratio.

4. AFM analysis of the interaction of DNA with pre-incubated tetramers 1 and 2: AFM images of the adducts of DNA with pre-incubated **1**·$[PF_6]_4$ or **2**·$[PF_6]_4$ are shown in Figure S15 at a 30 min time-point. A very low density of plasmids, in short L and OC forms, was found on the mica surface after treatment with pre-incubated **1**·$[PF_6]_4$ (Fig. S15a), similarly to that observed without tetramer incubation (Fig. S14a, b). This is consistent with the ^{1}H NMR studies that showed that after 24 h of incubation only ca. 37% of the **1**·$[PF_6]_4$ tetramers decompose (Fig. S9). In fact, the majority of the remaining intact tetranuclear molecules **1** can still bind effectively to the DNA, decreasing its negative charge and therefore preventing its binding to the Mg^{2+}-treated mica substrate. As a consequence, the few plasmids attached to the surface (Fig. S15a) should correspond to DNA molecules that did not interact with tetramer **1**. This was proven by analyzing their average heights – 0.76 ± 0.09 nm (n=3) for OC and 0.73 ± 0.07 nm (n=9) for L plasmids – which are compatible with the heights measured for the corresponding free DNA forms (Figs. 3a-b, main script).

The conformation of the plasmids did not change significantly after treatment with incubated tetramer **2**·$[PF_6]_4$ (Fig S15b), for which AFM images closely resemble those of the free plasmid control samples (Fig. 3a, main script). This is consistent with the fact that tetramer **2**·$[PF_6]_4$ decomposes completely to $[Os_2(\eta^6$-*p*-cym$)_2(\mu^2$-OH$)_3]^+$ dimers and free prz linkers after 24 h, as shown by ^{1}H NMR studies (Figs. 2 and S9), thus confirming the lack of a strong interaction of this biologically-inactive species with DNA. As expected, the height of the DNA plasmids in Fig S15b is also very close to that of the control samples with values of 0.81 ± 0.08 nm (n= 16) and 0.8 ± 0.1 nm (n= 16) measured for the OC and the L forms, respectively. We noticed that a few of the OC plasmids appeared to be partially coiled. These data might indicate a certain degree of interaction between DNA and the monocationic Os^{II} hydroxido-bridged dimers, although such interaction appears to occur to a lesser extent than in the case of Os^{II} tetramers **1** and **2**, which yielded DNA adducts at least twice as high.

REFERENCES

[1] A. F. A. Peacock, A. Habtemariam, S. A. Moggach, A. Prescimone, S. Parsons, P. J. Sadler, *Inorg. Chem.* **2007**, *46*, 4049-4059.

[2] *CrysAlisPro.* **2007**, *171*, 5.

[3] G. M. Sheldrick, *Acta. Cryst.* **1990**, *A46*, 467-473.

[4] G. M. Sheldrick, *SHELX97* **1997**, Programs for Crystal Structure Analysis (Release 97-92); University of Göttingen, Germany.

[5] V. Vichai, K. Kirtikara, *Nat. Protocols* **2006**, *1*, 1112-1116.

[6] a) B. Nordén, F. Tjerneld, *FEBS Letters* **1976**, *67*, 368-370; b) C. Hiort, B. Norden, A. Rodger, *J. Am. Chem. Soc.* **1990**, *112*, 1971-1982.

[7] S. Chasovskikh, A. Dritschilo, *Appl. Surf. Sci.* **2002**, *188*, 481-485.

[8] a) J. Gómez-Segura, S. Caballero, V. Moreno, M. J. Prieto, A. Bosch, *J. Inorg. Biochem..* **2009**, *103*, 128-134; b) E. Amat, M. J. Prieto, V. Moreno, *Int. J. Pharm.* **2004**, *270*, 75-82.

[9] I. Horcas, R. Fernández, J. M. Gómez-Rodríguez, J. Colchero, J. Gómez-Herrero, A. M. Baró, *Rev. Sci. Instrum.* **2007**, *78*, 013705.

[10] Frisch, M. J.; et al. Gaussian 03, revision D 0.1; Gaussian Inc.: Wallingford CT, **2004**.

[11] a) A. D. Becke, *J. Chem. Phys.* **1993**, *98*, 5648-5652; b) C. Lee, W. Yang, R. G. Parr, *Phys. Rev. B* **1988**, *37*, 785-789.

[12] P. J. Hay, W. R. Wadt, *J. Chem. Phys.* **1985**, *82*, 270-283.

[13] A. D. McLean, G. S. Chandler, *J. Chem. Phys.* **1980**, *72*, 5639-5648.

[14] M. Cossi, N. Rega, G. Scalmani, V. Barone, *J. Comput. Chem.* **2003**, *24*, 669-681.

[15] a) M. E. Casida, C. Jamorski, K. C. Casida, D. R. Salahub, *J. Chem. Phys.* **1998**, *108*, 4439-4449; b) R. E. Stratmann, G. E. Scuseria, M. J. Frisch, *J. Chem. Phys.* **1998**, *109*, 8218-8224.

[16] W. R. Browne, N. M. O'Boyle, J. J. McGarvey, J. G. Vos, *Chem. Soc. Rev.* **2005**, *34*, 641-663.

[17] N. M. O'Boyle, J. G. Vos, GaussSum, Dublin City University. Available at http://gaussum.sourceforge.net, **2005**.

[18] R. O. Gould, C. L. Jones, T. A. Stephenson, D. A. Tocher, *J. Organomet. Chem.* **1984**, *264*, 365-378.

[19] a) B. L. Yu, Y. Chen, C. Ouyang, H. Y. Huang, L. N. Ji, H. Chao, *Chem. Commun.* **2013**, *49*, 810-812; b) I. Turel, J. Kljun, F. Perdih, E. Morozova, V. Bakulev, N. Kasyanenko, J. A. W. Byl, N. Osheroff, *Inorg. Chem.* **2010**, *49*, 10750-10752; c) A. Yadav, T. Janaratne, A. Krishnan, S. S. Singhal, S. Yadav, A. S. Dayoub, D. L. Hawkins, S. Awasthi, F. M. MacDonnell, *Mol. Cancer. Ther.* **2013**, *12*, 643-653; d) D. A. Lutterman, A. Chouai, Y. Liu, Y. Sun, C. D. Stewart, K. R. Dunbar, C. Turro, *J. Am. Chem. Soc.* **2008**, *130*, 1163-1170; e) T. Wilson, P. J. Costa, V. Félix, M. P. Williamson, J. A. Thomas, *J. Med. Chem.* **2013**, *56*, 8674-8683; f) K. K. Patel, E. A. Plummer, M. Darwish, A. Rodger, M. J. Hannon, *J. Inorg. Biochem.*, **2002**, *91, 220-229.*

[20] a) J. Wang, S. L. Higgins, B. S. Winkel, K. J. Brewer, *Chem. Commun.* **2011**, *47*, 9786-9788; b) A. M. Palmer, J. D. Knoll, C. Turro, *Dalton Trans.* **2015**, *44*, 3640-3646; c) A. M. Palmer, S. J. Burya, J. C. Gallucci, C. Turro, *ChemMedChem.* **2014**, *9*, 1260-1265; d) S. J. Burya, A. M. Palmer, J. C. Gallucci, C. Turro, *Inorg. Chem.* **2012**, *51*, 11882-11890.

[21] F. Moreno-Herrero, J. Colchero, A. M. Baró, *Ultramicroscopy* **2003**, *96*, 167-174.

[22] a) C. Srinivasan, J. Lee, F. Papadimitrakopoulos, L. K. Silbart, M. Zhao, D. J. Burgess, *J. Mol. Ther.* **2006**, *14*, 192-201; b) E. Delain, A. Fourcade, J. C. Poulin, A. Barbin, D. Coulaud, E. Lecam, E. Paris, *Microsc. Microanal. M.* **1992**, *3*, 457-470; c) C. Bustamante, J. Vesenka, C. L. Tang, W. Rees, M. Guthold, R. Keller, *Biochemistry* **1992**, *31*, 22-26.
